# Adaptive Evolution and Distinct Mutation Signatures of Full‐Length HBV Quasispecies in HBeAg‐Negative Chronic Hepatitis B

**DOI:** 10.1002/mbo3.70175

**Published:** 2025-11-27

**Authors:** Changhui Wu, Fengwei Liu, Xiao Li, Xiaojin Li, Hui Li, Sihang Zhang, Xiaohui Yan, Taicheng Zhou, Jia Wei

**Affiliations:** ^1^ Kunming Medical University Kunming Yunnan China; ^2^ Department of Infectious Diseases and Hepatology the Affiliated Hospital of Yunnan University Kunming Yunnan China; ^3^ Central Laboratory the Affiliated Hospital of Yunnan University Kunming Yunnan China

**Keywords:** genetic diversity, HBeAg‐negative chronic hepatitis B, hepatitis B virus, quasispecies, viral evolution

## Abstract

The evolutionary profile of hepatitis B virus (HBV) quasispecies may influence the clinical course of chronic hepatitis B (CHB), but few studies have characterized quasispecies according to hepatitis B e antigen (HBeAg) status. In this study, we analyzed 289 full‐length HBV clones from 19 treatment‐naïve CHB patients with long‐term infection (> 10 years), comprising nine HBeAg‐positive and ten HBeAg‐negative, using molecular cloning and Sanger sequencing. Compared with HBeAg‐positive patients, HBeAg‐negative patients displayed higher quasispecies diversity (mean intrapatient sequence divergence 1.09% vs. 0.44%) and more complex phylogenetic structures. They also exhibited a greater number of positively selected sites, with 70.8% located within known *T*‐ or *B*‐cell epitope regions, predominantly in the surface (S), polymerase (Pol), and *X* regions. Classical basal core promoter (BCP) and precore (PreC) mutations were detected in 52.8% of HBeAg‐negative clones, often coexisting with wild‐type strains. In patients lacking these classical BCP/preC mutations but showing sustained viremia, intrahost recombination was observed. Moreover, overlapping reading frames, particularly +1 frameshifts in Pol/S region, demonstrated asymmetric distribution patterns. In patients harboring deletion mutations, intact quasispecies were also maintained. Collectively, these findings reveal multiple adaptive strategies that sustain HBV replication and immune escape in HBeAg‐negative patients, providing mechanistic insights for disease monitoring and therapeutic interventions.

## Graphical abstract

HBeAg‐negative chronic hepatitis B harbors a highly heterogeneous HBV quasispecies shaped by region‐specific selection. Adaptive strategies underpinning viral persistence include: BCP/PreC mutations supporting replication, PreS/S mutations enabling immune evasion, and overlapping Pol/S mutations maintaining viral fitness. These findings provide insights for disease monitoring and therapy.

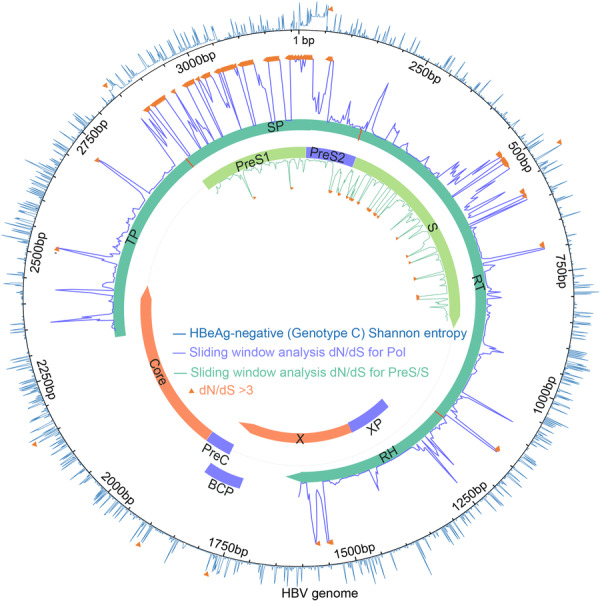

## 1 Introduction

The natural history of chronic HBV infection comprises five dynamic phases defined by hepatitis B surface antigen (HBsAg) and hepatitis B e antigen (HBeAg) status, viral load, and liver inflammation, reflecting host–virus interactions (Jeng et al. 2023). Despite substantial progress in antiviral therapy, a functional cure for chronic hepatitis B remains elusive (Chen et al. 2025). Among these phases, HBeAg‐negative chronic hepatitis B (CHB) has become the predominant clinical phenotype in many HBV‐endemic regions (Guardiola Arévalo et al. 2017). This phase is characterized by persistent or fluctuating viral replication and recurrent hepatic inflammation, often asymptomatic, which may silently progress to fibrosis, cirrhosis, and hepatocellular carcinoma (Yao et al. 2022; Alexopoulou 2014). This complicate early patient identification and poses challenges for treatment decisions and long‐term prognosis (Bonino et al. 2022).

HBeAg, a secreted viral protein, acts as an immune tolerogen facilitating HBV persistence. Seroconversion and HBeAg loss reflect the establishment of partial immune control, which imposes selective pressure on the virus and shapes its evolutionary trajectory (Wu et al. 2011; Kuipery et al. 2020). However, the molecular mechanisms regulating HBV replication following HBeAg seroconversion remain not fully understood. HBV replication is inherently error‐prone due to the lack of proofreading by its reverse transcriptase, generating a genetically heterogeneous quasispecies population (Hao et al. 2015; Domingo et al. 2012). This genetic diversity underpins viral adaptability, immune escape, and pathogenesis, which in turn drive disease progression, modulate treatment responses, and ultimately shape clinical outcomes (Domingo et al. 2025; Revill et al. 2020).

Most previous studies have focused on quasispecies diversity within selected genomic regions—such as the precore/core (preC/C), X (HBx), pre‐surface/surface (PreS/S), and polymerase (Pol) regions (Bayliss et al. 2017; Li et al. 2024; Ruan et al. 2018; Wu et al. 2021) or during HBeAg seroconversion (Wu et al. 2011). However, fragment‐based analyses are limited in capturing co‐occurring or compensatory mutations, particularly within the extensively overlapping open reading frame (ORF) organization, thereby restricting insights into viral evolution and clinical relevance (Valaydon and Locarnini 2017). In contrast, full‐length genome analysis provides an integrated view of the mutational landscape, encompassing regulatory regions, recombinant events, and genome‐wide interactions. Although our previous work characterized full‐length HBV quasispecies in patients with coexistent HBsAg and anti‐HBs (Zhou et al. 2017), comparable studies examining HBeAg status‐based full‐length HBV quasispecies in chronic hepatitis B patients remain scarce.

Considering these limitations, we analyzed plasma‐derived full‐length HBV sequences (genotypes B, C, and I) obtained via molecular cloning and Sanger sequencing from treatment‐naïve chronic hepatitis B patients in persistent HBeAg‐positive or ‐negative phases. This study aimed to comprehensively characterize genome‐wide mutation patterns and quasispecies diversity in relation to HBeAg serostatus. By delineating HBV evolutionary trajectories and immune escape mechanisms, our findings provide a framework for enhanced molecular diagnostics and the development of tailored therapeutic strategies in CHB management.

## 2 Methods

### 2.1 Study Population

We conducted a cross‐sectional study of 19 treatment‐naïve CHB patients, including nine HBeAg‐positive and ten HBeAg‐negative individuals, at the Affiliated Hospital of Yunnan University, China. Newly recruited participants consisted of the entire HBeAg‐negative group (*n* = 10) and three additional HBeAg‐positive patients. The remaining six HBeAg‐positive and one HBeAg‐negative patients had been previously included in a study on HBsAg/anti‐HBs coexistence (Zhou et al. 2017) and were reanalyzed here to serve as part of the comparison cohort. Inclusion criteria were HBsAg positivity for more than 10 years, serum HBV DNA ≥ 10^4^ IU/mL, and ALT > 40 IU/mL. Exclusion criteria included coinfection with HIV, HCV, or HDV, autoimmune liver disease, or pregnancy. All patients met the eligibility criteria and were classified according to HBeAg status. The study protocol was approved by the Institutional Review Board of the Affiliated Hospital of Yunnan University and conducted in accordance with the Declaration of Helsinki.

### 2.2 Biochemical, HBV Serological, and Imaging Analysis

Serum biochemical parameters were measured using an automated chemistry analyzer (Beckman Coulter, USA), and HBV serological markers were measured via an Abbott Architect immunoassay system (Abbott Laboratories, USA). HBV DNA levels were quantified using the Cobas TaqMan HBV Test on the Cobas TaqMan 48 Analyzer (Roche Diagnostics, Germany). Liver fibrosis was evaluated by transient elastography (FibroScan), and cirrhosis was diagnosed according to guideline‐recommended criteria (Lampertico et al. 2017), based on ultrasound findings and clinical evidence of decompensation.

### 2.3 HBV Genome Cloning and Sequencing

Total DNA was extracted from 200 µL of each patient plasma sample using the QIAamp MinElute Virus Spin Kit (Qiagen, Germany) according to the manufacturer's protocol and eluted in 50 µL of Elution Buffer AVE. Full‐length HBV genome was amplified by PCR in a 50 µL reaction, containing 8–10 µL of DNA template, 1 µL of high‐fidelity LA Taq DNA polymerase (Takara, Dalian, China), 10 µL of 10× LA PCR buffer, 16 µL of dNTPs (2.5 mM each), 2 µL of forward primer (5′‐TTTTTCACCTCTGCCTAATCA‐3′, nt 1821–1841), and 2 µL of reverse primer (5′‐AAAAAGTTGCATGGTGCTGG‐3′, nt 1825–1806), with nuclease‐free water to a final volume of 50 µL. The PCR profile was as follows: initial denaturation at 95°C for 3 min; 30 cycles of three successive sets of 10 cycles each (denaturation at 94°C for 40 s, annealing at 60°C for 90 s, and extension at 68°C for 3, 5, or 7 min for the first, second, and third set, respectively); final extension at 72°C for 7 min. PCR products were purified using a QIAquick PCR Purification Kit (Qiagen, Germany), ligated into the pGEM‐T Easy Vector (Promega, USA), and transformed into DH5α *Escherichia coli* cells (TaKaRa Boi, Japan) using a heat‐shock method at 42°C for 90 s, followed by recovery in 600 µL of LB medium at 37°C with shaking for 1 h. Positive clones were screened by PCR, and plasmid DNA was extracted using a Plasmid Mini Kit I (OMEGA Bio‐Tek, USA) and sequencing was performed on an ABI 3730 automated sequencer (Applied Biosystems, USA) using six primers as previously described (Zhou et al. 2017; Yang et al. 2015).

### 2.4 Sequence Analysis

Clone selection prioritized full‐length coverage and high sequence quality while maintaining a minimum number of representative sequences per sample to capture major intra‐host variants and reduce selection bias. On average, 15 clones were sequenced per patient. Sequence quality was assessed using CodonCode Aligner v11.0.2 with default quality trimming parameters. HBV full‐genome sequences were assembled using SeqMan (v7.1.0). Sequences were aligned with reference strains (Genotype B: GenBank AY167097; Genotype C: GenBank AB033556; Genotype I: GenBank GU357844) using MUSCLE 3.8.31. HBV genotyping was conducted using the NCBI Viral Genotyping tool (http://www.ncbi.nlm.nih.gov/projects/genotyping/form-page.cgi).

### 2.5 Quasispecies Analysis

Nucleotide differences across full‐length genomes were calculated using the distance method implemented in MEGA 11 (Tamura et al. 2021). Viral quasispecies heterogeneity was assessed using two indices: complexity and diversity. Complexity was quantified as standardized Shannon entropy (Sn) (Zhang et al. 2024), reflecting both the number and distribution of variants within a sample. For each nucleotide position (nt1 to nt3215), complexity was calculated as: $\mathrm{Sn}=-\Sigma_{i\in [A,T,C,G,-]}\;\mathrm{pi}\;\ln\mathrm{pi}/\ln N$, where pi represents the relative frequency of a nucleotide or deletion at each position, and N is the total number of clones analyzed (Zagordi et al. 2012). Complexity distribution plots were generated using the R (version 4.5.0) circlize package (version 0.4.16) (Gu et al. 2014). Diversity indices, including the mean genetic distance (d), synonymous substitutions per synonymous site (dS), and nonsynonymous substitutions per nonsynonymous site (dN), were calculated using MEGA 11. Sequence variants within the S, C, P, and X regions were analyzed to identify nucleotide and amino acid substitutions. Amino acid mutations were annotated according to a systematic review of experimentally validated HBV T‐cell epitopes (Wu et al. 2022), with potential B‐cell epitopes predicted using the Immune Epitope Database (IEDB, https://iedb.org/).

### 2.6 Phylogenetic Analysis

A phylogenetic tree was constructed based on full‐length HBV genome sequences, with Genotype D (AB090269) designated as the outgroup for rooting and comparative analysis. The best‐fit evolutionary model was determined using the Model Selection feature in MEGA11 under the Bayesian Information Criterion (BIC), which identified GTR + I + G as the optimal. The phylogenetic tree was constructed using PhyML 3.1 with the maximum likelihood method, and node support was assessed via 1000 bootstrap replicates (Guindon et al. 2010). Phylogenetic trees were visualized using FigTree v1.4.4 (available at http://tree.bio.ed.ac.uk).

### 2.7 Recombination Analysis

Putative recombination events were screened using RDP4 (v.4.101) with six algorithms (RDP, GENECONV, Bootscan, MaxChi, Chimaera, and SiScan) under default settings (Martin et al. 2015). A clear intrahost recombination was detected in patient P14. Using RDP4 results, the major and minor parental quasispecies sequences, together with one reference genotype C and one genotype H sequence from NCBI, were selected for BootScan analysis using SimPlot to validate intrahost recombination. Due to variable breakpoints among clones, two representative regions—non‐recombinant (nt 500–2400) and recombinant (spanning nt 2800–3215 and nt 1–300)—were manually defined for maximum‐likelihood phylogenetic analysis, incorporating reference sequences from genotypes B, D, and subgenotypes C1–C6.

### 2.8 Selection Pressure Analysis

Sequences containing insertions, deletions, or premature stop codons were excluded from analysis. The dN/dS ratio was estimated using the branch‐model in PAML 4.9j (Yang 2007). Sliding window Nei–Gojobori distance was calculated with SWAAP1.0.2 (http://www.bacteriamuseum.org/SWAAP/SwaapPage.htm). Selection pressure was quantified using the variable ω, where *ω* < 1, *ω* = 1, and *ω* > 1 indicate purifying selection, neutral evolution, and positive selection, respectively. Positive selection codons were identified using PAML's site‐models (M1 vs. M2, M7 vs. M8) and evaluated via likelihood ratio tests (LRTs). Posterior probabilities were estimated using the empirical Bayes method (Yang 2005).

### 2.9 Statistical Analysis

Statistical analyses were conducted using SPSS Statistics version 26 (IBM Corp., Armonk, NY, USA). Data are presented as means ± standard deviations (SDs) for normally distributed data or medians with interquartile ranges (IQRs) for non‐normally distributed variables. Categorical variables are expressed as counts and percentages. Complexity and polymorphisms were evaluated using independent samples *t*‐tests or Wilcoxon rank‐sum tests. Sn values at each nucleotide position were compared using paired *t* tests. Differences in point mutations and amino acid substitutions were evaluated using chi‐square tests or Fisher's exact tests. To correct for multiple comparisons, false discovery rate (FDR)‐adjusted *p*‐values were calculated for chi‐square tests using the p.adjust function in R (version 4.5.0). Statistical significance was defined as *p* < *0.05*.

## 3 Results

### 3.1 Patient Demographics and Clinical Characteristics

To evaluate quasispecies according to HBeAg status, we first compared baseline clinical characteristics between groups. Demographic and clinical parameters are summarized in Table 1. Compared with the HBeAg‐positive cohort, the HBeAg‐negative patients (all male, median age 48 years) were significantly older and exhibited higher ALT and AST levels, elevated liver stiffness, and lower HBsAg titers, HBV DNA levels, and serum albumin (all *p* < 0.05). In contrast, the HBeAg‐positive group included both sexes, and showed lower liver stiffness. Only two HBeAg‐negative patients (P11 and P17) presented with clinical features of decompensated cirrhosis. The remaining HBeAg‐negative patients (including P14, P15, and P16) were asymptomatic and identified incidentally during routine health examinations or follow‐up for unrelated conditions.

**Table 1:** Patient demographics and clinical features.

Characteristic	HBeAg‐positive (*n* = 9)	HBeAg‐negative (*n* = 10)	*p* value	OR (95% CI)
Age (y)	33.89 ± 10.38	50 ± 10.23	< 0.001	1.22(1.04–1.43)
Males: females	5:4	10:0	0.033	—
HBV DNA (log_10_ IU/mL)	8.07 ± 0.65	6.76 ± 0.77	< 0.001	0.02(0.00–0.61)
HBsAg (log_10_ IU/mL)	4.59 ± 0.65	3.14 ± 0.44	0.001	0.10 (0.01–0.66)
ALT (U/L)	82(47–115)	172(130–250)	0.038	1.00 (1.00–1.01)
AST (U/L)	44(40–51)	117(85–129)	0.005	1.00(1.00–1.01)
TBIL (μmol/L)	13(11.0, 20.3)	25(20.0, 72.0)	0.017	1.12 (0.96–1.32)
ALB (g/L)	44.7(42.7–49.4)	36.9(23.0–41.5)	0.005	0.66(0.41–1.06)
AFP (ng/mL)	6.67(5.05–8.18)	5.94(4.25–28.0)	0.711	1.00(0.99–1.01)
Live Elasticity (Kpa)	8.75(6.8–9.95)	15.4(11.1–37.9)	0.015	1.73 (0.88–3.41)
Cirrhosis, *n* (%)	0	2(20%)	0.047	—
Alcohol consumption, *n* (%)	1(11.1%)	5(50%)	0.141	—

*Note:* Data are shown as *n* (%) for categorical variables, median (IQR) or mean ± SD for continuous variables.

Abbreviations: AFP, alpha‐fetoprotein; ALB, albumin; ALT, alanine aminotransferase; AST, aspartate transaminase; HBsAg, hepatitis B surface antigen; HBV DNA, hepatitis B virus deoxyribonucleic Acid; tBil, total bilirubin.

### 3.2 HBeAg‐Negative Patients Exhibit Greater Quasispecies Diversity

**Figure 1:**
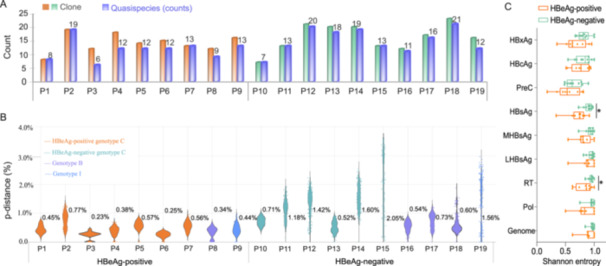
Quasispecies clone counts, intrahost genetic distances, and region‐specific complexity. (A) The number of clones and quasispecies (haplotypes) per patient. (B) Mean nucleotide divergence (p‐distance) of intrahost quasispecies relationships. Values adjacent to violin plots represent the mean p‐distance. (C) Gene‐specific complexity of HBV quasispecies, quantified by Shannon entropy. (*p < 0.05). HBcAg, hepatitis B core antigen; HBsAg, hepatitis B surface antigen; HBxAg, hepatitis B X antigen; LHBsAg, large hepatitis B surface antigen; MHBsAg, middle hepatitis B surface antigen; Pol, polymerase; PreC, precore region; RT, reverse transcriptase.

**Figure 2:**
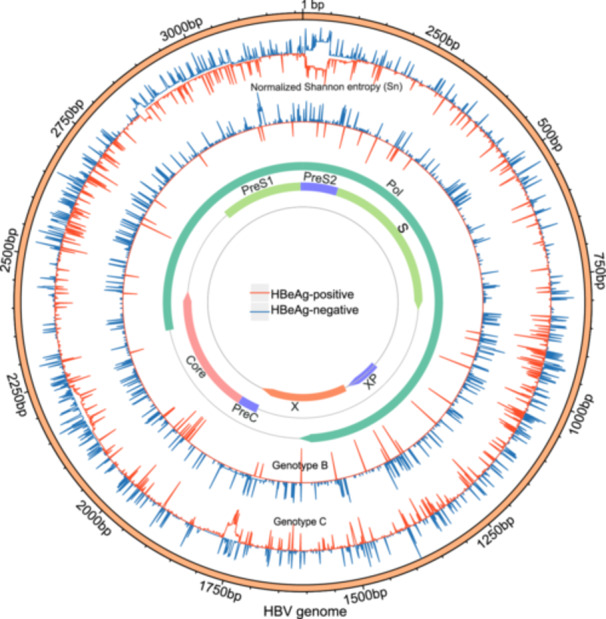
Nucleotide complexity (Shannon entropy) across the full‐length HBV genome (nt 1–3215). The insertions were discarded.

A total of 289 full‐length clone sequences were obtained, with an average of 15 clones per patient. The number of clones and quasispecies per patient is summarized (Figure 1A). The mean intrahost genetic distance was 0.44% ± 0.17% in HBeAg‐positive and 1.09% ± 0.54% in HBeAg‐negative patients, with the latter showing significantly higher diversity (*p* = 0.004; Figure 1B). Quasispecies complexity at the nucleotide level was evaluated for major HBV genomic regions, including large HBsAg (LHBsAg), middle HBsAg (MHBsAg), HBsAg, HBxAg, Hepatitis B core antigen (HBcAg), Pol, reverse transcriptase (RT) region, and the core promoter (Figure 1C). The HBeAg‐negative group exhibited significantly greater complexity in the RT and HBsAg regions. Position‐specific Shannon entropy (Sn) values across the full‐length HBV genome (nt 1–3215) were calculated to visualize nucleotide complexity in genotypes B and C (Figure 2). The HBeAg‐negative group presented higher nucleotide complexity than the HBeAg‐positive group across most genomic regions, except for the HBxAg region (*p* < 0.001; Table S1).

**Figure 3:**
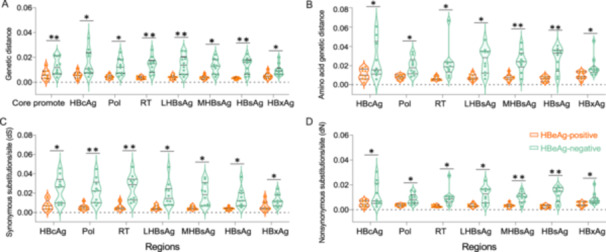
Quasispecies diversity of HBV across major genomic regions. (A) Mean nucleotide genetic distance (d_nt); (B) Mean amino acid genetic distance (d_aa); (C) Synonymous substitution rate (dS); (D) Nonsynonymous substitution rate (dN). (*p < 0.05, **p < 0.01).

Quasispecies diversity also differed significantly between groups. The HBeAg‐negative cohort presenting higher nucleotide genetic divergence at both the nucleotide and amino acid levels, alongside elevated synonymous (dS) and nonsynonymous substitution (dN) rates across all genomic regions (Figure 3).

### 3.3 Greater Phylogenetic Complexity in HBeAg‐Negative Patients

**Figure 4:**
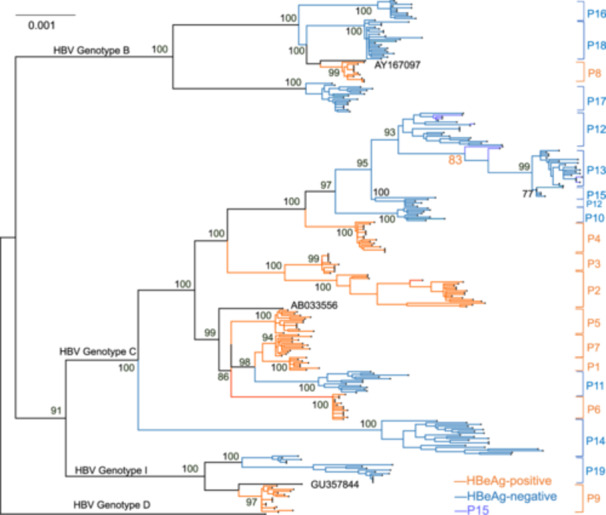
Phylogenetic tree of HBV genomes. The genotype D sequence (AB090269) was used as an outgroup, with genotypes B (AY167097), C (AB033556), and I (FJ023667) included as references. Patient sequences are labeled with identifiers P1‐P19. Bootstrap support values are shown at key nodes.

Maximum‐likelihood phylogenetic trees were constructed from full‐length HBV clones to compare phylogenetic relationships between groups. Overall, HBeAg‐negative patients more complex phylogenetic structures than HBeAg‐positive patients, as quantified by the mean phylogenetic branch length (mean ± SE: 0.00285  ±  0.00019 vs. 0.00158  ±  0.00015, *p* < 0.001) (Figure 4). All sequences formed well‐supported monophyletic clusters (> 70% bootstrap), which clustered by genotypes B, C, and I. While most patients presented high intrahost sequence homogeneity, sporadic interpatient clustering occurred within the HBeAg‐negative group. Notably, sequences from patients P12 and P15 clustered within the same subbranch, suggesting convergent evolution driven by common selective pressures. Sequences from patient P14 formed a distinct, well‐supported monophyletic cluster separate from other subbranches. This clustering was supported by full‐length nucleotide divergence analysis, which revealed greater than 1% nucleotide divergence among these patients, highlighting the genetic complexity and evolutionary dynamics of this subgroup.

### 3.4 Identification of an Intrahost C Recombinant in the Pol/S Overlap Region

**Figure 5:**
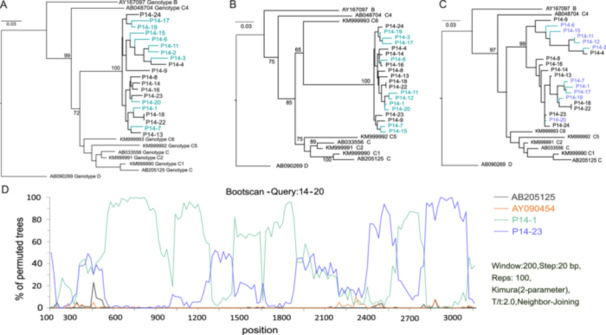
Phylogenetic and recombination analyses of HBV quasispecies from patient P14. (A) Maximum likelihood tree of full‐length HBV clones with genotype references. (B) Tree of the major parental region. (C) Tree of the recombinant region (nt 2800–3215 to 1–300). (D) SimPlot analysis of representative clones and reference sequences, confirming recombination breakpoints.

Recombination analysis of full‐length HBV quasispecies from patient P14 identified 11 recombination events (Table 2). Each event was confirmed by at least four detection methods (*p* < *0.05*) and was supported by bootstrap values exceeding 70% for the parental relationships. The recombination breakpoints clustered mainly between nucleotide positions 292 and 2990 (Table 2). Phylogenetic trees constructed from different genomic regions (Figure 5A–C) exhibited distinct topologies, confirming the region‐specific nature of these recombination events. SimPlot analysis (Figure 5D) confirmed the presence of recombinant fragments, corroborating results from multiple detection methods. Collectively, these data confirmed intrahost recombination in patient P14, with no detection of intergenotypic recombination.

**Table 2:** Recombination events and breakpoint analysis in patient P14.

Potential recombinant	Major parent	Minor parent	Breakpoint 1	Breakpoint 2	Bootstrap peak	Methods *(p‐values)*							Event
						1	2	3	4	5	6	7	
P14‐3	P14‐8	P14‐4	375–2759	2760–3215, 1–376	100%								1
P14‐6	P14‐15	P14‐16	1284–2713	2714–3215, 1–1283	98.4%								1
P14‐7	P14‐3	P14‐16	1009–2483	2484–3215, 1–1008	99.3%								1
P14‐12, P14‐11	P14‐17	P14‐1	292–1286	1287–3215, 1–291	100%								2
P14‐15	P14‐13	P14‐6	1165–3183	3184–3215, 1–1164	99.0%								1
P14‐17, P14‐1	P14‐6	P14‐16	364–2990	2991–3215, 1–363	99.6%								2
P14‐19, P14‐17	P14‐24	P14‐22	2262–2995	2996–3215, 1–2261	100%								2
P14‐20	P14‐1	P14‐23	582–2389	2390–3215, 1–581	100%								1

*Note:* Bootstrap peak values in the table represent the maximum support obtained from Bootscan analysis for each recombinant segment. Breakpoints shown were calculated by RDP and indicate the positions of recombination events with high confidence (threshold ≥ 75%). The statistical support (***p‐values**)* for the recombination events,as detected by the seven methods in RDP v.4.101, is color‐coded as follows: white filling, non‐significant; grey, *p* < 0.05; black, *p* < 0.01. The methods are numbered as: 1  =  RDP, 2 = Geneconv, 3 = Bootscan, 4 = Maxchi, 5 = Chimera, 6 = Siscan, 7 = 3Seq).

### 3.5 HBV Genes Under Purifying Selection

**Figure 6:**
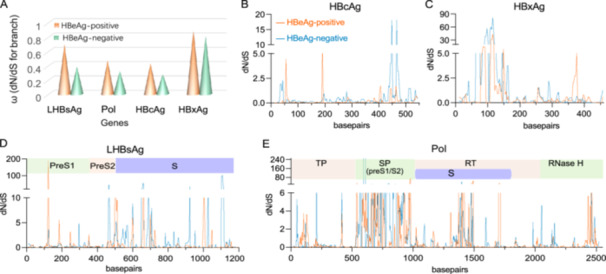
Distinct selective pressure patterns across major HBV genes. **(**A) Overall dN/dS ratios per gene estimated by the PAML branch model. Likelihood ratio tests (LRTs) were used to compare selection pressures between groups. (B‐E) dN/dS ratios were calculated using a sliding window approach (window: 15 nt, step: 3 nt) based on the Nei–Gojobori method. dN/dS, nonsynonymous to synonymous substitution ratio.

To evaluate evolutionary pressures on HBV genes, we compared overall dN/dS ratios between the two groups using branch‐model analyses and assessed local adaptation signals through sliding‐window analysis. Branch‐model likelihood ratio tests (LRTs) yielded non‐significant results across all HBV proteins (X: –66.12, Pol: –161.79, HBcAg: –65.68, LHBsAg: –42.22; all *p* > 0.05), indicating no difference in selective pressures between cohorts. All estimated ω values were below 1, indicating purifying selection in both cohorts (Figure 6 A). Sliding window analysis, however, revealed focal regions with dN/dS > 1 in four coding genes (Figure 6B‐E). Notably, in the genomic region where the Polymerase and Surface genes overlap via a +1 frameshift, we observed asymmetric selection pressures. Specifically, elevated dN/dS values were predominantly clustered in the Surface gene reading frame (approximately nt 500–1000, corresponding to the PreS1/PreS2 domains), while the overlapping Polymerase reading frame (corresponding to part of the RT domain) showed comparatively fewer signals of positive selection. This contrasting pattern highlights the distinct evolutionary constraints acting on each gene within the same nucleotide sequence.

### 3.6 Positive Selection Sites Identified in HBeAg‐Negative Patients

To further characterize adaptive evolution, we applied PAML site models (M8) to detect positively selected codons in HBV proteins. Significant evidence of positive selection was observed in LHBsAg, HBxAg, HBcAg, and Pol among HBeAg‐negative patients (LRTs, *p* < 0.05; Table 3 and Supplementary Table S2). In total, 24 codons under positive selection (posterior probability > 0.95, Bayes empirical Bayes analysis) were identified among 162 full‐length HBV clones and were represent in quasispecies from 90% of HBeAg‐negative patients. Notably, 17 (70.8%) of these sites mapped within known T‐cell or B‐cell epitopes. The positively selected residues included positions 188, 195, 221, 300 and 374 in LHBsAg; 5, 36, 87, 94, 127 and 130 in HBxAg; 84 and 97 in HBcAg; and 46, 312, 340, and 485 in Pol (Table 3). These results indicate that immune pressure underpins localized adaptive evolution in immunogenic and functionally critical regions of the HBV genome in HBeAg‐negative patients.

**Table 3:** Codons in the HBV genes that were under positive selection.

Gene	Codon position	Wide type amino acid (B, C, I genotype)	Posterior probability^d^	Omega^e^	Subjects and clones at each site		FDR‐adjusted *p* value^f^
					HBeAg‐positive 127(9)	HBeAg‐negative 162(10)	
LHBsAg	4	W	0.995	3.673 ± 0.715	0	15 (4)	< 0.001
	188	V^a^,^b^	0.968	3.596 ± 0.836	0	17 (4)	< 0.001
	195	L^a^,^b^	0.994	3.671 ± 0.718	0	63 (7)	< 0.001
	221	V, T,V^a^,^b^	0.992	3.664 ± 0.730	0	46 (4)	< 0.001
	300	T, I, T^c^	1.0	3.686 ± 0.690	15 (1)	64 (8)	< 0.001
	374	F, Y, Y^a^	0.990	3.659 ± 0.740	0	23 (6)	< 0.001
HBxAg	5	L, V, L^a^	0.974	3.439 ± 0.696	49 (3)	97 (9)	< 0.001
	36	A, P, D^a^	0.998	3.508 ± 0.573	60 (4)	79 (9)	0.813
	39	P, S, S	0.998	3.508 ± 0.572	1 (1)	21 (4)	< 0.001
	40	A, P, S	0.996	3.415 ± 0.729	0	17 (2)	< 0.001
	87	G, Q, M^a^	0.984	3.470 ± 0.647	31 (3)	67 (7)	0.0032
	94	H^a^	0.978	3.451 ± 0.678	12 (1)	46 (5)	< 0.001
	127	V, I, I^a^,^b^,^c^	1	3.513 ± 0.562	0	104 (8)	< 0.001
	130	K^a^,^b^,^c^	0.999	3.511 ± 0.567	46 (6)	113 (9)	< 0.001
HBcAg	84	L^b^	0.953	2.233 ± 0.579	0	40 (4)	< 0.001
	97	I^a^	0.957	2.237 ± 0.571	7 (2)	83 (8)	< 0.001
Pol	46	L,L,P^a^	0.999	2.516 ± 0.145	1 (1)	55 (5)	< 0.001
	184	L	0.978	2.481 ± 0.281	0 (0)	13 (3)	0.001
	312	S, A, A^a^	0.986	2.495 ± 0.238	16 (2)	23 (5)	0.763
	340	C, T, S^a^	0.967	2.497 ± 0.229	3 (1)	112 (9)	< 0.001
	485	N,N,Q^a^	0.980	2.485 ± 0.268	1 (1)	14 (2)	0.0030
	682	L, L, M	0.995	2.509 ± 0.183	21 (2)	56 (6)	< 0.001
	710	I	0.986	2.494 ± 0.238	47 (3)	51 (8)	0.416
	841	R, K, R	0.994	2.508 ± 0.187	79 (9)	76 (6)	0.014

^a^ HLA I T cell epitopes.

^b^ HLA II T cell epitopes.

^c^ B cell epitopes.

^d^ The number of clones with positive selection (the number of affected patients).

^e^ Display the results of HBeAg negative group.

^f^ The number of clones with positive selection in HBeAg‐positive vs HBeAg‐negative, chi‐square testing, *p* values were adjusted using the False Discovery Rate (FDR) method.

### 3.7 Increased Mutation Rates in HBeAg‐Negative Patients

#### 3.7.1 BCP/PreC/C Region Mutations

**Figure 7:**
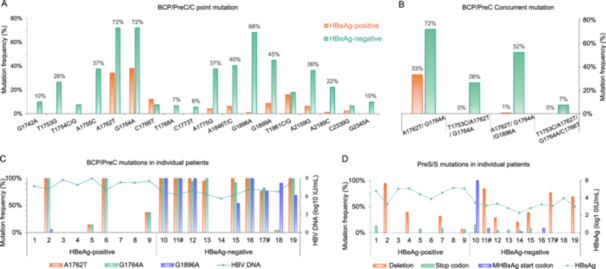
Mutation frequencies in BCP/preC/C and S regions of HBV. (A) Hotspot mutations in the BCP/preC/C regions. (B) Co‐occurrence of mutations in the BCP and preC regions. (C) BCP/preC mutations per patient in relation to HBV DNA levels. (D) Mutations in the S region per patient in relation to HBsAg titers. # Patients with decompensated cirrhosis.

Compared with the wild‐type reference sequence, 14 clinically relevant mutations were identified in the BCP and preC/C regions, which exhibited significantly different frequencies between the HBeAg‐negative and HBeAg‐positive patients (Figure 7A). The preC stop codon mutation G1896A was markedly more frequent in the HBeAg‐negative group (68.1% vs. 0.8%, *p* < 0.001). The classical BCP double mutation A1762T/G1764A (72.2% vs. 33.1%, *p* < 0.001) and the A1762T/G1764A/G1896A triple mutation (52.4% vs. 0.8%, *p* < 0.001) were also enriched in HBeAg‐negative patients (Figure 7B). Two additional variants, A2159G (36.4% vs. 6.3%, *p* < 0.001) and A2189C (22.2% vs. 0.8%, *p* < *0.05*), were also significantly enriched in this group. Detailed statistical comparisons are provided (Table S3).

Intrahost quasispecies analysis revealed that wild‐type variants predominated in HBeAg‐positive patients, whereas mutant strains predominated in HBeAg‐negative patients (Figure 7C). Within the HBeAg‐negative cohort, recombinant strain P14 lacked BCP/preC mutations. In contrast, clones from P13 carried BCP mutations in the absence of the preC G1896A stop codon. These findings demonstrate a strong association between viral mutation profiles and HBeAg status, and underscore the remarkable genetic heterogeneity within hosts across key HBV genomic regions.

#### 3.7.2 Amino Acid Variations

Amino acid substitutions were identified across the HBcAg, HBxAg, LHBsAg, and RT regions. Among genotype C patients, 88 sites differed significantly between HBeAg‐negative and HBeAg‐positive groups (9 in HBcAg, 16 in HBxAg, 37 in LHBsAg, and 26 in the RT domain, *p* < 0.05, Table S4), including many known HLA class I/II‐restricted T‐cell or B‐cell epitopes. Within the “a” determinant of HBsAg, I126T (46.8% vs. 7.1%), M133T (41.5% vs. 1.0%), and G130N (5.3% vs. 0.0%) were more frequent in HBeAg‐negative clones. A similar increase in I126T frequency was observed in genotypes B and I (*p* = 0.003; Table S5). These findings demonstrate more pronounced amino acid variation in key immunogenic regions of HBeAg‐negative patients, consistent with immune‐driven viral diversification under reduced antigenic tolerance.

Known drug resistance‐associated mutations were detected exclusively in treatment‐naïve HBeAg‐negative patients across three HBV genotypes (Table S6) (Rahman and Mahmood 2025). Patient P18 (genotype B) harbored the rtN236T in most clones (78.3%). P19 (genotype I) carried rtA181T alongside the overlapping sW172* mutation, and harbored distinct clones with rtM204V. Patient P11 (genotype C) had a single clone with rtM204I. No resistance mutations were identified in HBeAg‐positive patients. These findings highlight that clinically relevant resistance mutations can emerge spontaneously within the quasispecies population, independent of antiviral therapy.

### 3.8 Genomic Insertions, Deletions, and Stop Codon Mutations

Structural variations were characterized by analyzing insertions, deletions, and stop codon mutations in patient‐derived clones (Table 4; Table S7). Log‐transformed HBsAg levels and PreS1/PreS2/S mutation frequencies per patient are shown in Figure 7D. Genomic deletions in HBeAg‐negative patients were predominantly located in the LHBsAg and Pol regions (30.9% vs. 23.6% in HBeAg‐positive patients). Conversely, deletions in the preC/C and HBx regions were more frequent in HBeAg‐positive individuals. Notably, in patients harboring deletion mutations, non‐deleted quasispecies coexisted within the same patient. Mutations affecting the preS2 start codon (ATG → ATA/GTG) were identified in 8.0% of the clones from four HBeAg‐negative patients, potentially impairing MHBsAg translation. Additionally, 12‐bp in‐frame insertions (nt 1631–1642) in the HBxAg region overlapping enhancer II were detected in clones from patient P16. These results underscore region‐specific structural variation patterns that may modulate viral protein expression and contribute to clinical heterogeneity between HBeAg‐positive and HBeAg‐negative patients.

**Table 4:** The number of deletion, insertion, and stop codon mutations observed in the HBV quasispecies.

Gene	Mutations	HBeAg‐positive (*n* = 127)	HBeAg‐negative (*n* = 162)	FDR‐adjusted *p* value*
Genome	Deletion	54 (42.5%)	50 (30.9%)	0.060
	Insertion	0	12 (7.4%)	0.0021
	Stop codon mutations	11 (8.7%)	123 (75.9%)	< 0.001
LHBsAg	Deletion	30 (23.6%)	50 (30.9%)	0.2173
	Stop codon mutations	4 (3.1%)	5 (3.1%)	1
	PreS2 Iniation codon mutations	0	13 (8.0%)	0.0012
HBxAg	Deletion	24 (18.9%)	1	<0.001
	Insertion	0	12 (7.4%)	0.0021
PreC/C	Deletion	18 (14.2%)	1	<0.001
	Stop codon mutations	4 (3.1%)	110 (67.9%)	<0.001
Pol	Deletion	30 (23.6%)	50 (30.9%)	0.2173
	Stop codon mutations	3 (2.4%)	8 (4.9%)	0.4022

## 4 Discussion

HBeAg‐negative CHB represents a common and clinically challenging phase of infection, characterized by fluctuating viremia, increased viral genetic heterogeneity, and variable disease activity (Guardiola Arévalo et al. 2017; Xie et al. 2022). Despite its clinical significance, comprehensive whole‐genome analyses of HBV quasispecies evolution in this subgroup remain limited. Our comparative analysis of full‐length HBV quasispecies between HBeAg‐negative and HBeAg‐positive patients revealed several key features of viral evolution. HBeAg‐negative patients exhibited substantially greater intrapatient quasispecies complexity and genetic diversity, along with evidence of recombination involving genotype C, a pattern of overall purifying selection accompanied by localized adaptive evolution, and positive selection within immune epitopes. We also documented a high prevalence of classical BCP/preC mutations, region‐specific deletions, and naturally occurring resistance variants in treatment‐naïve HBeAg‐negative individuals. Notably, wild‐type and mutant clones coexisted within the same patient. Together, these findings delineate a multifaceted model of HBV adaptation and persistence in HBeAg‐negative CHB, providing a framework for refining personalized clinical management.

Our comprehensive analysis of full‐length HBV quasispecies stratified by HBeAg status demonstrated substantially greater intrapatient complexity and higher genetic diversity in HBeAg‐negative patients compared with HBeAg‐positive individuals. The mean pairwise genetic distance across full‐length quasispecies was 1.09% in HBeAg‐negative patients versus 0.44% in HBeAg‐positive patients, with some individuals exhibiting distances of up to 2.0%. Although these values remain below the divergence typically defining HBV subgenotypes (4%–7.5%) (McNaughton et al. 2020), they clearly underscore the existence of complex, patient‐specific quasispecies variants. Importantly, this elevated diversity was evident not only across the entire genome but also within major functional regions, including LHBsAg, HBcAg, Pol (encompassing the RT domain), and HBxAg. These observations align with quasispecies theory, which posits that the balance between mutation and selection continuously shapes the viral population landscape, thereby enhancing adaptability under host‐immune and replication pressures (Domingo et al. 2025). Previous studies have similarly linked increased replication capacity with higher quasispecies diversity and complexity in distinct HBV regions, such as the precore (Li et al. 2024), PreS/S (Wu et al. 2021), HBX, PreC/C during seroconversion (Wu et al. 2011), and HBx in HBeAg‐negative patients (Cortese et al. 2021). This extensive diversity is reflected in the patient‐specific phylogenetic clustering described below.

Phylogenetic analysis revealed that HBV sequences clustered primarily by genotype, forming patient‐specific subclusters that reflect intrahost viral evolution. In several HBeAg‐negative patients (P14, P15, P19), quasispecies formed complexphylogenetic trees characterized by long branches, corresponding to intrahost genetic distances exceeding 1%. Similar extreme intrahost divergence—at times exceeding the genetic distance between different patients—has been reported in longitudinal studies of HBeAg‐positive patients and is likely driven by intense intrahost competition (Osiowy et al. 2006; Xie and Lu 2024).

Notably, HBeAg‐negative patient P14 formed an independent phylogenetically distinct cluster within genotype C that was distant from all known C1–C6 subgenotypes. This patient maintained high HBV DNA levels and low HBsAg titer despite lacking classical BCP/PreC mutations. Recombination analysis identified high‐confidence intragenotypic events in the Pol/S overlap region, which is subject to dual‐frame evolutionary constraints. These events were supported by multiple detection methods in RDP4 and confirmed by bootscan analysis with significant bootstrap support. Although the polymerase spacer domain was recognized as a mutational hotspot (Pley et al. 2022), the polymerase gene is generally a recombination cold spot (Tshiabuila et al. 2025), and functional variation in this region may affect polymerase activity and viral replication dynamics. This intrahost recombination may represent an adaptive strategy that alters HBsAg antigenicity or secretion while preserving replication‐competent polymerase function, potentially explaining the paradoxical phenotype of low HBsAg titers despite high viremia in this patient (Chen et al. 2013). Similar intrapatient recombination has been reported in HCV quasispecies (Sentandreu et al. 2008), illustrating genetic segment exchange at the quasispecies level as a possible evolutionary strategy at the quasispecies level.

To elucidate the evolutionary forces shaping HBV quasispecies diversity, we evaluated selection pressures across the viral genome using branch and site models in PAML. Branch‐model analyses revealed pervasive purifying selection across major viral genes (dN/dS < 1), reflecting the conservation of essential viral functions (Wu et al. 2021). Site‐model analyses identified 24 positively selected codons distributed across the LHBsAg, HBxAg, HBcAg, and Pol, of which 70.8% were located within known B‐ or T‐cell epitopes (Walker et al. 2022; Lago et al. 2024). This dual evolutionary strategy—genome‐wide purifying selection maintaining viral fitness coupled with localized positive selection enabling immune escape—likely underlies the high quasispecies complexity and persistence observed in HBeAg‐negative CHB (Dolan et al. 2018).

Two key mutations, G1896A and A1762T/G1764A, modulate HBeAg expression and viral replication by introducing a premature stop codon in the preC region (Li et al. 2024) and enhancing pregenomic RNA synthesis in BCP region (Yang et al. 2023). In our cohort, both alterations were highly prevalent among HBeAg‐negative patients, consistent with their established roles in sustaining viral replication following HBeAg loss (Yang et al. 2023; Wu et al. 2014). Previous longitudinal studies of genotype C infection have shown that these variants increase in during HBeAg seroconversion (Wu et al. 2011). Their further increased prevalence after seroconversion suggests that these mutations may continue to accumulate or be positively selected within the quasispecies population after transition to the HBeAg‐negative phase. This pattern aligns with previous reports documenting a high prevalence of A1762T/G1764A mutations in patients with HBsAg/anti‐HBs coexistence (Zhou et al. 2017). Furthermore, the coexistence of wild‐type and mutant strains within hosts suggests the possibility of transient reversion, consistent with reports of HBeAg reappearance in a subset of patients (Chen et al. 2022; Lee et al. 2021). Additionally, we also detected an A2159G substitution (P87S/R) in the core coding region, which may influence capsid stability and HBeAg expression (Zhu et al. 2024). By contrast, deletions in the preC/C and HBx regions were more frequent in HBeAg‐positive patients, underscoring the phase‐dependent nature of HBV structural variation during chronic infection (Yang et al. 2015; Kumar Rajput 2020).

Within the X gene, we identified three notable nucleotide changes in HBeAg‐negative individuals: T1753C/G, A1762T, and G1764A corresponding to amino acid substitutions I127T/M, K130M and V131I in the C‐terminal HBx. These sites reside in the region where the BCP and HBx coding sequences overlap and may influence both transcriptional regulation and viral protein function, and have been implicated in hepatocarcinogenesis (Pu et al. 2022). In patient P16, who had a > 30‐year history of genotype B infection, we detected a novel 12‐bp in‐frame insertion (nt1631–1642) within a regulatory hotspot spanning enhancer II and the core promoter upstream of the BCP. Similar structural variations (SVs) have been reported between nt1500 and 2000 and may modulate viral transcription and persistence (Fujiwara et al. 2018; Fujiwara 2021). The prolonged infection history in this patient may have provided a unique evolutionary context favoring the emergence of such regulatory insertions. Notably, codons 127 and 130 were also identified as positively selected sites (BEB > 0.99), suggesting that adaptive pressure may have contributed to the retention of these variants.

HBeAg‐negative patients demonstrated complex quasispecies evolution primarily driven by structural variation, constrained by overlapping reading frames, and shaped by immune‐driven adaptation. Deletions in PreS1/PreS2, premature stop codons, and PreS2 start codon mutations can impair HBsAg secretion, potentially promoting oncogenic processes through endoplasmic reticulum stress and genomic instability (Wu et al. 2021; Pollicino et al. 2012; Lin et al. 2020; Teng et al. 2020). In our cohort, PreS/S deletions (30.9%) and PreS2 start codon mutations (8.0%) were associated with lower serum HBsAg levels, consistent with previous studies linking these defects to disease progression (Lin et al. 2021). Notably, patients with PreS/S deletions retained non‐deleted quasispecies, which can be explained by the complement–interference mechanism (Domingo et al. 2012): deleted genomes rely on intact genomes for replication (complementation), while their truncated proteins may interfere with full‐length genome function (interference), thereby maintaining viral diversity and overall fitness. Furthermore, amino acid substitutions in the HBsAg “a” determinant (I126T/S, G130N, M133T) were detected at relatively high frequencies in HBeAg‐negative patients. These variants, which alter B‐cell epitope, occurred at frequencies comparable to those reported in individuals with coexisting HBsAg and anti‐HBs (Zhou et al. 2017), suggesting they represent an alternative mechanism of immune evasion (Inuzuka et al. 2018) with potential implications for vaccine escape.

Beyond structural changes, HBeAg‐negative patients exhibited higher quasispecies complexity, with positively selected codons enriched in known T‐ and B‐cell epitopes, many of which were restricted by common HLA supertypes (HLA‐A02, A24, DRB1) (Wu et al. 2022). These epitopes represented targets of CD8⁺ T cell‐mediated selection, which can drive viral escape by altering HLA binding or antigen presentation (Lumley et al. 2018). Although individual host HLA genotypes were not assessed in this study, the overlap between positively selected sites and immunodominant epitopes underscores the role of immune‐driven pressures in shaping intrahost HBV evolution.

Sliding‐window analysis of the Pol/S overlapping region revealed asymmetric selection, with higher dN/dS ratios in the polymerase spacer compared with PreS, consistent with the “mutational buffer” hypothesis described in previous studies (Chen et al. 2013; Pavesi 2019; Zaaijer et al. 2007). This structurally permissive spacer domain can accommodate immune‐driven nonsynonymous mutations while minimizing deleterious effects on the overlapping S region, thereby enabling viral adaptation under immune pressure without compromising antigenicity or replication (Pollicino et al. 2012). These findings collectively underscore a critical balance between functional constraints and adaptive evolution, contributing to the elevated quasispecies complexity and immune escape potential observed in HBeAg‐negative CHB.

Resistance‐associated mutations (rtA181T, rtM204V/I, rtN236T) were identified in the RT region of treatment‐naïve HBeAg‐negative patients, accounting for 39.6% of 53 clones derived from three individuals. This observation is consistent with accumulating evidence that such variants can arise naturally in untreated individuals, especially those infected with genotype C (Mokaya et al. 2021). These findings underscore the importance of resistance testing when clinically indicated, thereby guiding personalized antiviral therapy strategies.

This study provides a comprehensive characterization of full‐length HBV quasispecies in treatment‐naïve HBeAg‐positive and HBeAg‐negative CHB patients using cloning and Sanger sequencing. This approach captured both intra‐ and interhost variation, enabling a detailed assessment of viral adaptive evolution, overlapping gene constraints, and their clinical associations. Our identification of recombination is based solely on genetic and phylogenetic analyses from single‐timepoint sequences; thus, future studies incorporating longitudinal sampling and in vitro functional assays are needed to definitively establish its contribution to viral persistence.

## 5 Conclusion

HBeAg‐negative chronic hepatitis B is characterized by high intrahost quasispecies diversity shaped by region‐specific adaptive pressures. Mutations in the BCP/preC region sustain viral replication, while preS/S variations facilitate immune evasion. Overlapping Pol/S mutations balance replication efficiency with envelope integrity. In patients carrying deletion mutations, intact quasispecies were maintained, thereby preserving essential viral functions. Intrahost recombination may also provide an alternative mechanism for sustaining viral replication in the absence of BCP/preC mutations. Collectively, these genome‐wide adaptive strategies underpin HBV persistence and evolution in the absence of HBeAg, offering mechanistic insights that could refine disease monitoring and inform therapeutic intervention.

## Author Contributions

**Changhui Wu:** investigation, data curation, validation, writing – original draft, writing – review and editing. **Fengwei Liu:** methodology, investigation, data curation. **Xiao Li:** investigation, methodology, review. **Xiaojin Li:** investigation, data curation. **Hui Li:** resources, data curation. **Sihang Zhang:** investigation. **Xiaohui Yan:** resources, data curation. **Taicheng Zhou:** conceptualization, study design, methodology. **Jia Wei:** conceptualization, study design, review and editing.

## Ethics Statement

This study was approved by the Ethics Committee of Yunnan University Affiliated Hospital (Approval No. 20165023, No. 2022128). This study was in accordance with the Declaration of Helsinki.

## Conflicts of Interest

The authors declare no conflicts of interest.

## Supporting information


**Table S1:** Quasispecies complexity (Sn) of each nucleotide position (nt1 to 3215) between the patients with HBeAg positive and HBeAg negative. **Table S2:** Summary of positive selection analyses for genes LHBsAg, HBxAg, HBcAg and Pol. **Table S3:** Number of variations at Core Promote and pre‐C/Core region (nt1742‐2452) in present study. **Table S4:** Amino acid variations in HBcAg, HBxAg, LHBsAg and polymerase RT region of genotype C. **Table S5:** Amino acid variations in “a” epitope (aa124–aa147). **Table S6:** The patient numbers and mutation clone counts at drug‐resistant mutation sites. **Table S7:** Summary of deletion, insertion, and stop codon mutations in HBV quasispecies detected.

## Data Availability

The data that support the findings of this study have been deposited in the NCBI GenBank repository under the accession numbers PV526051–PV526236 (newly submitted in this study), KY470857–KY470872, and KY470921–KY471007 (previously submitted). Additional data are available from the corresponding author upon reasonable request.

## References

[mbo370175-bib-0001] Alexopoulou, A. 2014. “HBeAg Negative Variants and Their Role in the Natural History of Chronic Hepatitis B Virus Infection.” World Journal of Gastroenterology 20, no. 24: 7644. 10.3748/wjg.v20.i24.7644. 24976702 PMC4069293

[mbo370175-bib-0002] Bayliss, J. , L. Yuen , G. Rosenberg , et al. 2017. “Deep Sequencing Shows That HBV Basal Core Promoter and Precore Variants Reduce the Likelihood of HBsAg Loss Following Tenofovir Disoproxil Fumarate Therapy in HBeAg‐Positive Chronic Hepatitis B.” Gut 66, no. 11: 2013–2023. 10.1136/gutjnl-2015-309300. 27534671

[mbo370175-bib-0003] Bonino, F. , P. Colombatto , and M. R. Brunetto . 2022. “HBeAg‐Negative/Anti‐HBe‐Positive Chronic Hepatitis B: A 40‐Year‐Old History.” Viruses 14, no. 8: 1691. 10.3390/v14081691. 36016312 PMC9416321

[mbo370175-bib-0004] Chen, J. , D. Ji , J. Jia , et al. 2025. “Functional Cure With new Antiviral Therapy for Hepatitis B Virus: A Systematic Review and Meta‐Analysis.” Hepatology International 19, no. 4: 773–795. 10.1007/s12072-025-10873-9. 40528088 PMC12287141

[mbo370175-bib-0005] Chen, P. , Y. Gan , N. Han , et al. 2013. “Computational Evolutionary Analysis of the Overlapped Surface (S) and Polymerase (P) Region in Hepatitis B Virus Indicates the Spacer Domain in P Is Crucial for Survival.” PLoS One 8, no. 4: e60098. 10.1371/journal.pone.0060098. 23577084 PMC3618453

[mbo370175-bib-0006] Chen, Q. Y. , H. H. Jia , X. Y. Wang , et al. 2022. “Analysis of Entire Hepatitis B Virus Genomes Reveals Reversion of Mutations to Wild Type in Natural Infection, a 15 Year Follow‐Up Study.” Infection, Genetics and Evolution 97: 105184. 10.1016/j.meegid.2021.105184. 34902556

[mbo370175-bib-0007] Cortese, M. F. , C. González , J. Gregori , et al. 2021. “Sophisticated Viral Quasispecies With a Genotype‐Related Pattern of Mutations in the Hepatitis BX Gene of HBeAg‐Ve Chronically Infected Patients.” Scientific Reports 11, no. 1: 4215. 10.1038/s41598-021-83762-4. 33603102 PMC7892877

[mbo370175-bib-0008] Dolan, P. T. , Z. J. Whitfield , and R. Andino . 2018. “Mechanisms and Concepts in RNA Virus Population Dynamics and Evolution.” Annual Review of Virology 5, no. 1: 69–92. 10.1146/annurev-virology-101416-041718. PMC13283306 30048219

[mbo370175-bib-0009] Domingo, E. , B. Martínez‐González , P. Somovilla , et al. 2025. “A General and Biomedical Perspective of Viral Quasispecies.” RNA 31, no. 3: 429–443. 10.1261/rna.080280.124. 39689947 PMC11874995

[mbo370175-bib-0010] Domingo, E. , J. Sheldon , and C. Perales . 2012. “Viral Quasispecies Evolution.” Microbiology and Molecular Biology Reviews 76, no. 2: 159–216. 10.1128/MMBR.05023-11. 22688811 PMC3372249

[mbo370175-bib-0011] Fujiwara, K. 2021. “Novel Genetic Rearrangements in Hepatitis B Virus: Complex Structural Variations and Structural Variation Polymorphisms.” Viruses 13, no. 3: 473. 10.3390/v13030473. 33809245 PMC8000817

[mbo370175-bib-0012] Fujiwara, K. , K. Matsuura , K. Matsunami , E. Iio , and S. Nojiri . 2018. “Characterization of Hepatitis B Virus With Complex Structural Variations.” BMC Microbiology 18: 202. 10.1007/978-3-030-71165-8_15. 30509169 PMC6276219

[mbo370175-bib-0013] Gu, Z. , L. Gu , R. Eils , and M. Schlesner , and B. Brors . 2014. “Circlize” Implements and Enhances Circular Visualization in R. 10.1093/bioinformatics/btu393 24930139

[mbo370175-bib-0014] Guardiola Arévalo, A. , R. Gómez Rodríguez , M. Romero Gutiérrez , et al. 2017. “Characteristics and Course of Chronic Hepatitis B e Antigen‐Negative Infection.” Gastroenterología y Hepatología (English Edition) 40, no. 2: 59–69. 10.1016/j.gastrohep.2016.11.002. 28007350

[mbo370175-bib-0015] Guindon, S. , J.‐F. Dufayard , V. Lefort , M. Anisimova , W. Hordijk , and Ob Gascuel . 2010. “New Algorithms and Methods to Estimate Maximum‐Likelihood Phylogenies: Assessing the Performance of PhyML 3.0.” Systematic Biology 59, no. 3: 307–321. 10.1093/sysbio/syq010. 20525638

[mbo370175-bib-0016] Hao, R. , K. Xiang , Y. Peng , et al. 2015. “Naturally Occurring Deletion/Insertion Mutations Within HBV Whole Genome Sequences in HBeAg‐Positive Chronic Hepatitis B Patients Are Correlated With Baseline Serum HBsAg and HBeAg Levels and Might Predict a Shorter Interval to HBeAg Loss and Seroconversion During Antiviral Treatment.” Infection, Genetics and Evolution: Journal of Molecular Epidemiology and Evolutionary Genetics in Infectious Diseases 33: 261–268. 10.1016/j.meegid.2015.05.013. 25976382

[mbo370175-bib-0017] Inuzuka, T. , Y. Ueda , S. Arasawa , et al. 2018. “Expansion of Viral Variants Associated With Immune Escape and Impaired Virion Secretion in Patients With HBV Reactivation After Resolved Infection.” Scientific Reports 8, no. 1: 18070. 10.1038/s41598-018-36093-w. 30584239 PMC6305382

[mbo370175-bib-0018] Jeng, W. J. , G. V. Papatheodoridis , and A. Lok . 2023. “Hepatitis B.” Lancet 401, no. 10381: 1039–1052. 10.1016/S0140-6736(22)01468-4. 36774930

[mbo370175-bib-0019] Kuipery, A. , A. J. Gehring , and M. Isogawa . 2020. “Mechanisms of HBV Immune Evasion.” Antiviral Research 179: 104816. 10.1016/j.antiviral.2020.104816. 32387476

[mbo370175-bib-0020] Kumar Rajput, M. 2020. “Mutations and Methods of Analysis of Mutations in Hepatitis B Virus.” AIMS Microbiology 6, no. 4: 401–421. 10.3934/microbiol.2020024. 33364535 PMC7755589

[mbo370175-bib-0021] Lago, B. V. , M. M. Portilho , V. M. Mello , et al. 2024. “Genetic Variability of Hepatitis B Virus in Acute and in Different Phases of Chronic Infection in Brazil.” Scientific Reports 14, no. 1: 10742. 10.1038/s41598-024-60900-2. 38730249 PMC11087654

[mbo370175-bib-0022] Lampertico, P. , K. Agarwal , T. Berg , et al. 2017. “EASL 2017 Clinical Practice Guidelines on the Management of Hepatitis B Virus Infection.” Journal of Hepatology 67, no. 2: 370–398. 10.1016/j.jhep.2013.11.003. 28427875

[mbo370175-bib-0023] Lee, W. M. , W. C. King , H. L. A. Janssen , et al. 2021. “Hepatitis B e Antigen Loss in Adults and Children With Chronic Hepatitis B Living in North America: A Prospective Cohort Study.” Journal of Viral Hepatitis 28, no. 11: 1526–1538. 10.1111/jvh.13591. 34355475 PMC8622507

[mbo370175-bib-0024] Li, G. , D. Yang , X. Liu , et al. 2024. “Precore Mutation Enhances Viral Replication to Facilitate Persistent Infection Especially in HBeAg‐Negative Patients.” Virologica Sinica 39, no. 2: 319–330. 10.1016/j.virs.2024.03.003. 38492851 PMC11074699

[mbo370175-bib-0025] Lin, J. , J. Li , P. Xie , et al. 2021. “Hepatitis B Virus Middle Surface Antigen Loss Promotes Clinical Variant Persistence in Mouse Models.” Virulence 12, no. 1: 2868–2882. 10.1080/21505594.2021.1999130. 34738866 PMC8632123

[mbo370175-bib-0060] Lin, W.‐L. , J.‐H. Hung , and W. Huang . 2020. “Association of the Hepatitis B Virus Large Surface Protein With Viral Infectivity and Endoplasmic Reticulum Stress‐Mediated Liver Carcinogenesis.” Cells 9, no. 9: 2052. 10.3390/cells9092052. 32911838 PMC7563867

[mbo370175-bib-0026] Lumley, S. F. , A. L. McNaughton , P. Klenerman , K. A. Lythgoe , and P. C. Matthews . 2018. “Hepatitis B Virus Adaptation to the CD8+ T Cell Response: Consequences for Host and Pathogen.” Frontiers in Immunology 9: 1561. 10.3389/fimmu.2018.01561. 30061882 PMC6054973

[mbo370175-bib-0027] Martin, D. P. , B. Murrell , M. Golden , A. Khoosal , and B. Muhire . 2015. “RDP4: Detection and Analysis of Recombination Patterns in Virus Genomes.” Virus Evolution 1, no. 1: vev003. 10.1093/ve/vev003. 27774277 PMC5014473

[mbo370175-bib-0028] McNaughton, A. L. , P. A. Revill , M. Littlejohn , P. C. Matthews , and M. A. Ansari . 2020. “Analysis of Genomic‐Length HBV Sequences to Determine Genotype and Subgenotype Reference Sequences.” Journal of General Virology 101, no. 3: 271–283. 32134374 10.1099/jgv.0.001387 PMC7416611

[mbo370175-bib-0029] Mokaya, J. , T. I. Vasylyeva , E. Barnes , M. A. Ansari , O. G. Pybus , and P. C. Matthews . 2021. “Global Prevalence and Phylogeny of Hepatitis B Virus (HBV) Drug and Vaccine Resistance Mutations.” Journal of Viral Hepatitis 28, no. 8: 1110–1120. 10.1111/jvh.13525. 33893696 PMC8581767

[mbo370175-bib-0030] Osiowy, C. , E. Giles , Y. Tanaka , M. Mizokami , and G. Y. Minuk . 2006. “Molecular Evolution of Hepatitis B Virus Over 25 Years.” Journal of Virology 80, no. 21: 10307–10314. 10.1128/microbe.1.578.1. 17041211 PMC1641782

[mbo370175-bib-0031] Pavesi, A. 2019. “Asymmetric Evolution in Viral Overlapping Genes Is a Source of Selective Protein Adaptation.” Virology 532: 39–47. 10.1016/j.virol.2019.03.017. 31004987 PMC7125799

[mbo370175-bib-0032] Pley, C. , J. Lourenço , A. L. McNaughton , and P. C. Matthews . 2022. “Spacer Domain in Hepatitis B Virus Polymerase: Plugging a Hole or Performing a Role?” Journal of Virology 96, no. 9: e00051–22. 10.1128/jvi.00051-22. 35412348 PMC9093120

[mbo370175-bib-0033] Pollicino, T. , G. Amaddeo , A. Restuccia , et al. 2012. “Impact of Hepatitis B Virus (HBV) Pres/S Genomic Variability on HBV Surface Antigen and HBV DNA Serum Levels.” Hepatology 56, no. 2: 434–443. 10.1002/hep.25592. 22271491

[mbo370175-bib-0034] Pu, R. , W. Liu , X. Zhou , et al. 2022. “The Effects and Underlying Mechanisms of Hepatitis B Virus X Gene Mutants on the Development of Hepatocellular Carcinoma.” Frontiers in Oncology 12: 836517. 10.3389/fonc.2022.836517. 35223517 PMC8867042

[mbo370175-bib-0035] Rahman, Z. U. , and M. Mahmood . 2025. “Resistant Mutations Against Nucleot (S) Ide Analogues (NAs) in RT Domain of HBV Genome: A Review.” Viral Hepatitis Journal 31: 1–7. 10.4274/vhd.galenos.2025.2023-9-1.

[mbo370175-bib-0036] Revill, P. A. , T. Tu , H. J. Netter , L. K. W. Yuen , S. A. Locarnini , and M. Littlejohn . 2020. “The Evolution and Clinical Impact of Hepatitis B Virus Genome Diversity.” Nature Reviews Gastroenterology & Hepatology 17, no. 10: 618–634. 10.1038/s41575-020-0296-6. 32467580

[mbo370175-bib-0037] Ruan, L. , J. A. Hadden , and A. Zlotnick . 2018. “Assembly Properties of Hepatitis B Virus Core Protein Mutants Correlate With Their Resistance to Assembly‐Directed Antivirals.” Journal of Virology 92, no. 20: 10–1128. 10.1128/JVI. PMC6158430 30089690

[mbo370175-bib-0038] Sentandreu, V. , N. Jiménez‐Hernández , M. Torres‐Puente , et al. 2008. “Evidence of Recombination in Intrapatient Populations of Hepatitis C Virus.” PLoS One 3, no. 9: e3239. 10.1371/journal.pone.0003239. 18800167 PMC2528950

[mbo370175-bib-0039] Tamura, K. , G. Stecher , and S. Kumar . 2021. “MEGA11: Molecular Evolutionary Genetics Analysis Version 11.” Molecular Biology and Evolution 38, no. 7: 3022–3027. 10.1093/molbev/msab120. 33892491 PMC8233496

[mbo370175-bib-0061] Teng, C.‐F. , T.‐C. Li , H.‐Y. Huang , et al. 2020. “Next‐Generation Sequencing‐Based Quantitative Detection of Hepatitis B Virus Pre‐S Mutants in Plasma Predicts Hepatocellular Carcinoma Recurrence.” Viruses 12, no. 8: 796. 10.3390/v12080796. 32722114 PMC7472021

[mbo370175-bib-0040] Tshiabuila, D. , J. E. San , E. Wilkinson , et al. 2025. “Conserved Recombination Patterns Across Hepatitis B Genotypes: A Retrospective Study.” Virology Journal 22, no. 1: 220. 10.1186/s12985-025-02829-0. 40618165 PMC12229012

[mbo370175-bib-0041] Valaydon, Z. S. , and S. A. Locarnini . 2017. “The Virological Aspects of Hepatitis B.” Best Practice & Research Clinical Gastroenterology 31, no. 3: 257–264. 10.1016/j.bpg.2017.04.013. 28774407

[mbo370175-bib-0042] Walker, A. , T. Schwarz , J. Brinkmann‐Paulukat , et al. 2022. “Immune Escape Pathways From the HBV core(18–27) CD8 T Cell Response Are Driven by Individual HLA Class I Alleles.” Frontiers in Immunology 13: 1045498. 10.3389/fimmu.2022.1045498. 36439181 PMC9686862

[mbo370175-bib-0043] Wu, J. F. , Y. H. Ni , H. L. Chen , H. Y. Hsu , and M. H. Chang . 2014. “The Impact of Hepatitis B Virus Precore/Core Gene Carboxyl Terminal Mutations on Viral Biosynthesis and the Host Immune Response.” Journal of Infectious Diseases 209, no. 9: 1374–1381. 10.1093/infdis/jit638. 24273041

[mbo370175-bib-0044] Wu, S. , F. Imazeki , F. Kurbanov , et al. 2011. “Evolution of Hepatitis B Genotype C Viral Quasi‐Species During Hepatitis B e Antigen Seroconversion.” Journal of Hepatology 54, no. 1: 19–25. 10.1016/j.jhep.2010.06.018. 20932594

[mbo370175-bib-0045] Wu, Y. , Y. Ding , and C. Shen . 2022. “A Systematic Review of T Cell Epitopes Defined From the Proteome of Hepatitis B Virus.” Vaccines 10, no. 2: 257. 10.3390/vaccines10020257. 35214714 PMC8878595

[mbo370175-bib-0046] Wu, Y. , Z. Zhu , J. Wu , W. Bi , W. Xu , and X. D. Han . 2021. “Evolutionary Analysis of Pre‐S/S Mutations in HBeAg‐Negative Chronic Hepatitis B With HBsAg ≪ 100 IU/Ml.” Frontiers in Public Health 9: 633792. 10.3389/fpubh.2021.633792. 33981663 PMC8107265

[mbo370175-bib-0047] Xie, C. , and D. Lu . 2024. “Evolution and Diversity of the Hepatitis B Virus Genome: Clinical Implications.” Virology 598: 110197. 10.1016/j.virol.2024.110197. 39098184

[mbo370175-bib-0048] Xie, Y. , H. Ma , B. Feng , and G. Song . 2022. “Combining the HBcrAg Decline and Hbv Mutations Predicts Spontaneous HBeAg Seroconversion in Chronic Hepatitis B Patients During the Immune Clearance Phase.” Journal of Medical Virology 94, no. 6: 2694–2701. 10.1002/jmv.27545. 34951036

[mbo370175-bib-0049] Yang, D. , J. Zou , G. Guan , et al. 2023. “The A1762T/G1764A Mutations Enhance HBV Replication by Alternating Viral Transcriptome.” Journal of Medical Virology 95, no. 10: e29129. 10.1002/jmv.29129. 37772469

[mbo370175-bib-0050] Yang, Z. 2005. “Bayes Empirical Bayes Inference of Amino Acid Sites Under Positive Selection.” Molecular Biology and Evolution 22, no. 4: 1107–1118. 10.1093/molbev/msi097. 15689528

[mbo370175-bib-0051] Yang, Z. 2007. “PAML 4: Phylogenetic Analysis by Maximum Likelihood.” Molecular Biology and Evolution 24, no. 8: 1586–1591. 10.1093/molbev/msm088. 17483113

[mbo370175-bib-0052] Yang, Z. T. , S. Y. Huang , L. Chen , et al. 2015. “Characterization of Full‐Length Genomes of Hepatitis B Virus Quasispecies in Sera of Patients at Different Phases of Infection.” Journal of Clinical Microbiology 53, no. 7: 2203–2214. 10.1128/JCM.00068-15. 25926495 PMC4473231

[mbo370175-bib-0053] Yao, K. , J. Wang , L. Wang , et al. 2022. “Association of Anti‐HBC and Liver Inflammation in HBeAg‐Negative Chronic Hepatitis B Virus‐Infected Patients With Normal ALT and Detectable HBV DNA.” Journal of Medical Virology 94, no. 2: 659–666. 10.1002/jmv.27327. 34499353

[mbo370175-bib-0054] Zaaijer, H. L. , F. J. van Hemert , M. H. Koppelman , and V. V. Lukashov . 2007. “Independent Evolution of Overlapping Polymerase and Surface Protein Genes of Hepatitis B Virus.” Journal of General Virology 88, no. 8: 2137–2143. 10.1099/vir.0.82906-0. 17622615

[mbo370175-bib-0055] Zagordi, O. , M. Däumer , C. Beisel , and N. Beerenwinkel . 2012. “Read Length Versus Depth of Coverage for Viral Quasispecies Reconstruction.” PLoS One 7, no. 10: e47046. 10.1371/journal.pone.0047046. 23056573 PMC3463535

[mbo370175-bib-0056] Zhang, C. , S. An , R. Lv , et al. 2024. “The Dynamic Variation Position and Predominant Quasispecies of Hepatitis B Virus: Novel Predictors of Early Hepatocarcinoma.” Virus Research 341: 199317. 10.1016/j.virusres.2024.199317. 38242020 PMC10831745

[mbo370175-bib-0057] Zhou, T.‐C. , X. Li , L. Li , X.‐F. Li , L. Zhang , and J. Wei . 2017. “Evolution of Full‐Length Genomes of HBV Quasispecies in Sera of Patients With a Coexistence of HBsAg and Anti‐HBs Antibodies.” Scientific Reports 7, no. 1: 661. 10.1038/s41598-017-00694-8. 28386078 PMC5428874

[mbo370175-bib-0058] Zhu, C. , M. Tang , Y. Fu , et al. 2024. “Characterization of BCP/PreC/C Region Quasispecies in Treatment‐Naive Patients With Different Phases of HBV Infection Using Next‐Generation Sequencing.” International Journal of Medical Microbiology 315: 151619. 10.1016/j.ijmm.2024.151619. 38564936

