# Supplementary material for: Adaptive Evolution and Distinct Mutation Signatures of Full‐Length HBV Quasispecies in HBeAg‐Negative Chronic Hepatitis B
Source: Microbiologyopen. 2025 Nov 27;14(6):e70175. doi: 10.1002/mbo3.70175 (PMC12660055; doi:10.1002/mbo3.70175)
Supplement: Supplementary file 1 — Table S1: Quasispecies complexity (Sn) of each nucleotide position (nt1 to 3215) between the patients with HBeAg positive and HBeAg negative. Table S2: Summary of positive selection analyses for genes LHBsAg, HBxAg, HBcAg and Pol. Table S3: Number of variations at Core Promote and pre‐C/Core region (nt1742‐2452) in present study. Table S4: Amino acid variations in HBcAg, HBxAg, LHBsAg and polymerase RT region of genotype C. Table S5: Amino acid variations in “a” epitope (aa124–aa147). Table S6: The patient numbers and mutation clone counts at drug‐resistant mutation sites. Table S7: Summary of deletion, insertion, and stop codon mutations in HBV quasispecies detected. [file MBO3-14-e70175-s001.docx]

**Distinct mutation and evolutionary profiles of full-length genomes of HBV quasispecies in HBeAg-negative chronic hepatitis B**

Changhui Wu^1,2#^, Fengwei Liu^1,3,#^, Xiao Li^3^, Xiaojin Li^3^, Hui Li^2^, Sihang Zhang^1^, Xiaohui Yan^2^, Taicheng Zhou^3,*^, Jia Wei^2,3,*^

^1^ Kunming Medical University, Kunming, Yunnan, China

^2^ Department of Infectious Diseases and Hepatology, the Affiliated Hospital of Yunnan University, Kunming, Yunnan, China

^3^ Central Laboratory, the Affiliated Hospital of Yunnan University, Kunming, Yunnan, China

^#^ Changhui Wu and Fengwei Liu contributed equally to this study

^*^ Corresponding authors: Prof. Jia Wei, Emai: weijia19631225@163.com;

Dr. Taicheng Zhou, Emai: zhoutc@ynshhyy.com.

**Table S1** Quasispecies complexity (Sn) of each nucleotide position (nt1 to 3215) between the patients with HBeAg positive and HBeAg negative

| Region of variation | HBeAg positive(mean±SE) | HBeAg negative(mean±SE) | *p* value* |
| --- | --- | --- | --- |
| **Genotype C** | | | |
| Full length | 0.06973±0.0037 | 0.10557±0.0045 | <0.001 |
| LHBsAg | 0.05280±0.0051 | 0.09279±0.0066 | <0.001 |
| MHBsAg | 0.04649±0.0055 | 0.08681±0.0075 | <0.001 |
| HBsAg | 0.03938±0.0059 | 0.07438±0.0078 | <0.001 |
| Polymerase | 0.0723±0.0043 | 0.10087±0.0050 | <0.001 |
| ProC/C | 0.04968±0.0068 | 0.11236±0.0107 | <0.001 |
| RT region | 0.06949±0.0068 | 0.09685±0.0076 | <0.001 |
| HBXAg | 0.06606±0.0094 | 0.08511±0.0114 | 0.0674 |
| **Genotype B** | | | |
| Full length | 0.00734±0.0012 | 0.07856±0.0039 | <0.001 |
| MHBsAg | 0.00446±0.0013 | 0.06424±0.0055 | <0.001 |
| LHBsAg | 0.00438±0.0016 | 0.06398±0.0067 | <0.001 |
| HBsAg | 0.00423±0.0018 | 0.05250±0.0066 | <0.001 |
| Polymerase | 0.00657±0.0013 | 0.07902±0.0044 | <0.001 |
| ProC/C | 0.01026±0.0031 | 0.06581±0.0083 | <0.001 |
| RT region | 0.00573±0.0018 | 0.07463±0.0068 | <0.001 |
| HBxAg | 0.00783±0.0037 | 0.06755±0.0096 | <0.001 |
| **Genotype I** |  |  |  |
| Full length | 0.011051±0.0014 | 0.031143±0.0027 | <0.001 |
| MHBsAg | 0.009521±0.0020 | 0.027883±0.0042 | <0.001 |
| LHBsAg | 0.008250±0.0021 | 0.031344±0.0053 | <0.001 |
| HBsAg | 0.007741±0.0023 | 0.032104±0.0059 | <0.001 |
| Polymerase | 0.010154±0.0002 | 0.02495±0.0005 | <0.001 |
| ProC/C | 0.015055±0.0037 | 0.047733±0.0076 | 0.0001 |
| RT region | 0.010631±0.0021 | 0.032554±0.0048 | <0.001 |
| HBxAg | 0.012205±0.0041 | 0.033281±0.0068 | 0.0637 |
| * HBeAg-positive and HBeAg-negative CHB paired sample t-test at the same nucleotide site | | | |

**Table S2** Summary of positive selection analyses for genes LHBsAg, HBxAg, HBcAg and Pol

| Gene | LHBsAg | | HBxAg | | HBcAg | | Pol | |
| --- | --- | --- | --- | --- | --- | --- | --- | --- |
|  | HBeAg+ | HBeAg- | HBeAg+ | HBeAg- | HBeAg+ | HBeAg- | HBeAg+ | HBeAg- |
| Model 1 | | | | | | | | |
| lnL | -4028.35 | -6481.18 | -1723.61 | -2407.60 | -1847.14 | -3092.76 | -9517.68 | -13001.71 |
| dN/dS (p0) | 0.233 (0.66) | 0.083 (0.523) | 0.091 (0.373) | 0.093 (0.481) | 0.108 (0.743) | 0.159 (0.79) | 0.104 (0.691) | 0.129 (0.72) |
| dN/dS (p1) | 1.00 (0.341) | 1.00(0.476) | 1.00 (0.627) | 1.00(0.519) | 1.00(0.256) | 1.00 (0.208) | 1.00 (0.309) | 1.00 (0.283) |
| Model 2 | | | | | | | | |
| lnL | -4028.30 | -6457.84 | -1715.53 | -2388.24 | -1847.14 | -3089.69 | -9500.12 | -12980.95 |
| dN/dS (p0) | 0.253 (0.686) | 0.10 (0.51) | 0.0001 (0.445) | 0.164 (0.480) | 0.108 (0.744) | 0.193 (0.840) | 0.104 (0.682) | 0.144 (0.720) |
| dN/dS (p1) | 1.00 (0.305) | 1.000 (0.432) | 1.000 (0.543) | 1.00 (0.401) | 1.00 (0.188) | 1.00 (0.078) | 1.00 (0.315) | 1.00 (0.253) |
| dN/dS (p2) | 2.340 (0.008) | 3.392 (0.058) | 10.10 (0.012) | 3.87 (0.119) | 1.00 (0.068) | 1.826 (0.082) | 8.999 (0.003) | 3.329 (0.026) |
| 2ΔlnL(LRT) (2vs1) | 0.05 | 46.68 | 8.08 | 19.36 | 0.00 | 3.07 | 17.56 | 20.76 |
| *p* value | 0.975 | 77.304e-11 | 0.017 | 6.252e-05 | 1.00 | 0.2155 | 1.538e-04 | 3.105e-05 |
| Postitively selected sites* | - | 4W, 195L, 221T,300I,374F | 36A | 36P, 39S, 87Q, 127I, 130K | - |  | 615L, 803H, 841K | 46L, 312A, 340T,682M,710I 841R |
| Model 7 | | | | | | | | |
| lnL | -4028.47 | -6483.60 | -1723.73 | -2408.29 | -1847.29 | -3098.59 | -9520.53 | -13014.75 |
| p | 0.538 | 0.0261 | 0.031 | 0.025 | 0.231 | 0.427 | 0.199 | 0.269 |
| q | 0.552 | 0.022 | 0.012 | 0.015 | 0.467 | 0.784 | 0.324 | 0.431 |
| Model 8 | | | | | | | | |
| lnL | -4028.22 | -6458.41 | -1715.54 | -2388.51 | -1846.99 | -3089.72 | -9501.69 | -12983.59 |
| p | 0.849 | 0.1937 | 1.291 | 0.0371 | 0.291 | 6.116 | 0.197 | 0.56852 |
| q | 0.995 | 0.1882 | 0.819 | 0.0332 | 0.624 | 22.776 | 0.314 | 1.26642 |
| p0 | 0.978 | 0.9368 | 0.925 | 0.867 | 0.994 | 0.874 | 0.995 | 0.93136 |
| dN/dS (p1) | 2.193 (0.022) | 3.3286 (0.063) | 4.516 (0.075) | 3.739 (0.133) | 3.575(0.006) | 1.631 (0.125) | 7.270 (0.005) | 2.305 (0.069) |
| 2ΔlnL(LRT) (8vs7) | 0.25 | 45.75 | 8.19 | 19.78 | 0.3 | 8.87 | 18.84 | 31.16 |
| *p* value | 0.882 | 1.163e-10 | 0.017 | 5.068e-05 | 0.861 | 0.0118 | 8.109e-05 | 1.713e-7 |
| Postitively selected sites* | - | 4W, 188V, 195L, 221T, 300I, 374Y | 36A | 5L,36P, 39S, 40P, 87Q, 94H, 127I, 130K |  |  | 615I,803H, 841K | 46L,184L 312A, 340T, 485N, 682L, 710I, 841R |

*BEB>0.95

**Table S3** Number of variations at Core Promote and pre-C/Core region (nt1742-2452) in present study

| mutations | HBeAg positive（n=127） | | | | HBeAg negative（n=162） | | | |  | | | FDR adjusted |  |  |  |  |  |
| --- | --- | --- | --- | --- | --- | --- | --- | --- | --- | --- | --- | --- | --- | --- | --- | --- | --- |
|  | wild type | mutant type | | wild type | | mutant type | | | *p*-value | | *p*-value | | | |  |  |  |
| G1742A | 127 | 0 | | 146 | | 16(9.9%) | | <0.001 | | <0.001 | | | | | |  |  |
| T1753G | 127 | 0 | | 118 | | 43(26.5%) | | <0.001 | | <0.001 | | | | | |  |  |
| T1754C/G | 127 | 0 | | 150 | | 12(7.4%) | | <0.004 | | 0.006 | | | | | |  |  |
| A1755C | 127 | 0 | | 101 | | 61(37.6%) | | <0.001 | | <0.001 | | | | | |  |  |
| A1762T | 85 | 42(33.1%) | | 45 | | 117(72.2%) | | <0.001 | | <0.001 | | | | | |  |  |
| G1764A | 85 | 42(33.1%) | | 45 | | 117(72.2%) | | <0.001 | | <0.001 | | | | | |  |  |
| C1766T | 112 | 15(11.8%) | | 150 | | 12(7.4%) | | 0.283 | | 0.302 | | | | | |  |  |
| T1768A | 127 | 0 | | 151 | | 11(68%) | | 0.007 | | 0.009 | | | | | |  |  |
| C1773T | 127 | 0 | | 153 | | 9(5.6%) | | 0.018 | | 0.023 | | | | | |  |  |
| A1775G | 122 | 5(3.9%) | | 101 | | 61(37.6%) | | <0.001 | | <0.001 | | | | | |  |  |
| A1846T/C | 119 | 8(6.3%) | | 96 | | 66(40.7%) | | <0.001 | | <0.001 | | | | | |  |  |
| G1896A | 126 | 1(0.8%) | | 51 | | 111(68.5%) | | <0.001 | | <0.001 | | | | | |  |  |
| G1899A | 126 | 0 | | 89 | | 73(45.1%) | | <0.001 | | <0.001 | | | | | |  |  |
| T1961C/G | 107 | 20(15.7%) | | 133 | | 29(17.9%) | | 0.744 | | 0.744 | | | | | |  |  |
| A2159G | 121 | 8(6.3%) | | 103 | | 59(36.4%) | | <0.001 | |  | | | | | |  |  |
| A2189C | 126 | 1(0.8%) | | 126 | | 36(22.2%) | | <0.001 | |  | | | | | |  |  |
| C2339G | 124 | 3(2.4%) | | 151 | | 11(6.8%) | | 0.143 | | 0.164 | | | | | |  |  |
| G2345A | 127 | 0 | | 146 | | 16(9.9%) | | <0.001 | | 0.001 | | | | | |  |  |
| Combined mutations | | | | | | | | |  | | | | | | | | |
| A1762T/G1764A | 85 | | 42(33.1%) | | 45 | | 117(72.2%) | | <0.001 | |  | | |  |  |  |  |
| T1753C/A1762T/G1764A | 127 | | 0 | | 119 | | 43(26.5%) | | <0.001 | |  | | |  |  |  |  |
| A1762T/G1764A/G1896A | 126 | | 1(0.8%) | | 77 | | 85(52.4%) | | <0.001 | |  | | |  |  |  |  |
| T1753C/A1762T/G1764A/C1766T | 127 | | 0 | | 150 | | 12(7.4%) | | 0.002 | |  | | |  |  |  |  |
| FDR p-values were adjusted using the Benjamini-Hochberg method to control the false discovery rate (FDR). | | | | | | | | | | | | | | | | |  |

**Table S4** Amino acid variations in HBcAg, HBxAg, LHBsAg and polymerase RT region of genotype C

| Genes | Position | Mutations | HBeAg positive (99 clones) | HBeAg negative (94 clones) | Chi-square test | FDR- adjusted | Epitopes |
| --- | --- | --- | --- | --- | --- | --- | --- |
|  |  |  | mutant type | mutant type | *p* value | *p* value |  |
| HBcAg | 21 | S21A | 16(16.2%) | 17(18.1%) | 0.722 | 0.75 | HLA I/II T,B |
| HBcAg | 25 | V25I | 98(99.0%) | 78(83.0%) | <0.001 | <0.001 | HLA I/II T,B |
| HBcAg | 35 | S35A | 0 | 20(21.3%) | <0.001 | <0.001 | HLA II T, |
| HBcAg | 60 | V60L | 97(99.0%) | 58(61.7%) | <0.001 | <0.001 | HLA I/II T,B |
| HBcAg | 87 | S87G/R | 10(10.1%) | 49(52.1%) | <0.001 | <0.001 | HLA I/II T,B |
| HBcAg | 97 | I97L | 4(4.0%) | 13(13.8%) | 0.0213 | 0.268 | HLA I/II T,B |
| HBcAg | 101 | L101W | 0 | 20(21.3%) | <0.001 | <0.001 | HLA I/II T,B |
| HBcAg | 112 | R112G | 3(3.0%) | 1(1.1%) | 0.622 | 0.652 | HLA I/II T,B |
| HBcAg | 115 | V115A | 4(5.1%) | 1(1.1%) | 0.369 | 0.403 | HLA I/II T,B |
| HBcAg | 130 | P130S/T | 49(49.5%) | 13(13.8%) | <0.001 | <0.001 | HLA I/II T,B |
| HBcAg | 135 | P135Q | 1(1.0%) | 39(41.5%) | <0.001 | <0.001 | HLA I/II T,B |
| HBcAg | 153 | G153C | 3(3.0%) | 56(59.6%) | <0.001 | <0.001 | HLA I/II T,B |
| HBcAg | 180 | E180G | 15(15.2%) | 8(8.5%) | 0.155 | 0.178 | HLA II T,B |
| HBxAg | 5 | V5L/M | 49(50.5%) | 46(48.9%) | 0.938 | 0.947 | HLA I, B |
| HBxAg | 30 | V30L | 23(23.2%) | 38(40.4%) | 0.010 | 0.013 | HLA I, B |
| HBxAg | 32 | S32R | 0 | 90(95.7%) | <0.001 | <0.001 | HLA I, B |
| HBxAg | 36 | G36S/A/T | 49(50.5%) | 94(100%) | <0.001 | <0.001 | HLA I, B |
| HBxAg | 39 | P38S/L | 21(21.2%) | 2(2.1%) | <0.001 | <0.001 | B |
| HBxAg | 42 | L42S | 35(35.4%) | 93(98.9%) | <0.001 | <0.001 | B |
| HBxAg | 43 | S43P | 30(30.3%) | 0 | <0.001 | <0.001 | B |
| HBxAg | 47 | A47T | 15(15.2%) | 45(47.9%) | <0.001 | <0.001 | HLA I, B |
| HBxAg | 48 | N48D | 96(98.0%) | 88(93.6%) | 0.3214 | 0.3586 | HLA I, B |
| HBxAg | 61 | C61R/Y | 6(6.1%) | 0 | 0.0291 | 0.0355 | HLA I T,B |
| HBxAg | 64 | S64T | 12(12.1%) | 1(1.1%) | 0.0026 | 0.0035 | HLA I T,B |
| HBxAg | 86 | R86H/L/S | 38(38.4%) | 49(52.1%) | 0.0551 | 0.0663 | B |
| HBxAg | 95 | K95N | 14(14.1%) | 9(9.6%) | 0.3276 | 0.3618 | HLA I T,B |
| HBxAg | 101 | S101P/A | 15(15.2%) | 2(2.1%) | <0.0016 | 0.0022 | HLA I T,B |
| HBxAg | 116 | V116L | 15(15.2%) | 0 | <0.001 | <0.001 | HLA I/II T,B |
| HBxAg | 123 | L123S | 18(19.2%) | 39(41.5%) | <0.001 | <0.001 | HLA I/II T,B |
| HBxAg | 127 | I127T/M/F | 3(4.0%) | 59(62.8%) | <0.001 | <0.001 | HLA II T,B |
| HBxAg | 130 | K130M | 40(40.4%) | 64(68.1%) | <0.001 | <0.001 | HLA II T,B |
| HBxAg | 131 | V 131 I /T | 40(40.4%) | 73(77.7%) | <0.001 | <0.001 | HLA II T,B |
| HBxAg | 132 | Y132F | 80(81.8%) | 93(98.9%) | <0.001 | <0.001 | HLA II T,B |
| LHBsAg | 10 | K10Q | 65(65.7%) | 69(73.4%) | 0.2429 | 0.2768 |  |
| LHBsAg | 14 | T14S | 6(6.1%) | 0 | 0.0291 | 0.0355 |  |
| LHBsAg | 27 | D27G | 17(17.3%) | 61(64.9%) | <0.001 | <0.001 | HLA I/II, T,B |
|  |  | D27E | 0 | 14(8.6%) | <0.001 | <0.001 | HLA I T,B |
| LHBsAg | 35 | G35R | 8(8.1%) | 5(5.3%) | 0.4442 | 0.4756 |  |
| LHBsAg | 41 | P41S | 14(14.3%) | 0 | <0.001 | <0.001 | HLA II, T,B |
| LHBsAg | 51 | H51Q | 17(17.3%) | 47(50.0%)) | <0.001 | <0.001 | HLA II, T |
| LHBsAg | 54 | A54E | 50(50.5%) | 31(33.0%) | 0.0136 | 0.0174 |  |
| LHBsAg | 60 | A60V | 27(27.6%) | 78(83.0%) | <0.001 | <0.001 |  |
| LHBsAg | 62 | A62S | 17(17.3%) | 61(64.9%) | <0.001 | <0.001 | T (HLA II), |
| LHBsAg | 67 | F67L | 1(1.0%) | 11(11.7%) | 0.0021 | 0.0028 | T (HLA II), |
| LHBsAg | 73 | S73G | 49(49.0%) | 22(23.4%) | <0.001 | <0.001 | T (HLA I /II) |
| LHBsAg | 90 | A90V | 15(15.3%) | 0 | <0.001 | <0.001 | T (HLA I) |
| LHBsAg | 102 | G102R | 0 | 28(29.8%) | <0.001 | <0.001 |  |
| LHBsAg | 120 | M120T/V | 0 | 9(9.6%) | 0.0013 | 0.0018 | HLA I/II T, B |
| LHBsAg | 125 | T125S | 17(17.3%) | 53(56.4%) | <0.001 | <0.001 | HLA II T, B |
| LHBsAg | 130 | A130T | 1(1.0%) | 34(36.2%) | <0.001 | <0.001 | HLA II T, B |
| LHBsAg | 141 | F141C | 0 | 11(11.7%) | <0.001 | <0.001 | HLA II T, B |
| LHBsAg | 161 | I161V | 17(17.3%) | 0 | <0.001 | <0.001 | HLA II T, B |
| LHBsAg | 177 | N177S | 54(54.1%) | 5(5.3%) | <0.001 | <0.001 | HLA II |
| LHBsAg | 182 | F182L | 0 | 7(7.4%) | <0.0058 | 0.0077 | HLA I/II T, B |
| LHBsAg | 184 | G184R/K/T | 0 | 9(9.6)%) | 0.0012 | 0.0018 | HLA I/II T, B |
| LHBsAg | 188 | V188A | 0 | 16(17.0%) | <0.001 | <0.001 | HLA I/II T, B |
| LHBsAg | 195 | L195S | 8(8.2%) | 11(11.7%) | 0.3986 | 0.4311 | HLA I/II T, B |
| LHBsAg | 198 | R198K | 0 | 21(22.3%) | <0.001 | <0.001 | HLA I/II T, B |
| LHBsAg | 219 | A219V/T | 0 | 22(23.4%) | <0.001 | <0.001 | HLA I/II T, B |
| LHBsAg | 221 | T221V/A | 3(3.1%) | 23(24.5%) | <0.001 | <0.001 | HLA I/II T, B |
| LHBsAg | 223 | P223R | 4(4.1%) | 5(5.3%) | 0.7426 | 0.7642 | HLA I/II T, B |
| LHBsAg | 227 | L227S | 52(52.5%) | 21(22.3%) | <0.001 | <0.001 | HLA I, T, B |
| LHBsAg | 242 | T242I | 66(66.3%) | 4(4.3%) | <0.001 | <0.001 | HLA I/II T, B |
| LHBsAg | 250 | C250Y | 19(19.4%) | 0 | <0.001 | <0.001 | HLA I/II T |
| LHBsAg | 300 | I300V/T/G/S | 24(24.5%） | 58（61.7%) | <0.001 | <0.001 | HLA I/II T, B |
| LHBsAg | 301 | G301R/N | 0 | 78(83.0%) | <0.001 | <0.001 |  |
| LHBsAg | 307 | M307T | 1(1.0%) | 39(41.5%) | <0.001 | <0.001 | HLA I T, B |
| LHBsAg | 308 | F308Y | 1(1.0%) | 2(2.1%) | 0.6134 | 0.6502 | HLA I T, B |
| LHBsAg | 336 | L336Q | 15(15.3%) | 0 | <0.001 | <0.001 | HLA I/II T |
| LHBsAg | 349 | L349S | 12(12.2%) | 0 | <0.001 | <0.001 | HLA I/II T |
| LHBsAg | 368 | A368V | 65(65.3%) | 94(100%) | <0.001 | <0.001 | HLA I T |
| LHBsAg | 377 | P377Q | 12(12.2%) | 0 | <0.001 | <0.001 | HLA I T |
| LHBsAg | 378 | S378R | 0 | 21(22.3%) | <0.001 | <0.001 | HLA I T |
| LHBsAg | 381 | N381S | 20(20.4%) | 0 | <0.001 | <0.001 | HLA I T |
| LHBsAg | 382 | I382T | 0 | 5(5.3%) | 0.0259 | 0.0323 | HLA I T |
| LHBsAg | 384 | S384N | 17(17.3%) | 48(51.1%) | <0.001 | <0.001 | HLA I T |
| LHBsAg | 398 | V398A | 0 | 3(3.2%) | 0.1136 | 0.1338 | HLA I T |
| RT | 9 | H9Y | 17(17.3%) | 8(8.5%) | 0.0732 | 0.0872 | HLA I T |
| RT | 13 | N13H | 41(41.8%) | 77(81.9%) | <0.001 | <0.001 | HLA I T |
| RT | 16 | I16T | 0 | 7(7.4%) | <0.001 | <0.001 | HLA I T |
| RT | 18 | R18K/N | 0 | 11(11.7%) | <0.001 | <0.001 | HLA I T |
| RT | 38 | T38A | 13(13.3%) | 1(1.1%) | 0.0013 | 0.0019 | HLA I T |
| RT | 50 | S50P | 0 | 12(12.8%) | <0.001 | <0.001 | HLA I T |
| RT | 53 | S53N | 0 | 16(17.0 | <0.001 | <0.001 | HLA I T |
| RT | 55 | H55R | 3(3.1%) | 26(27.7%) | <0.001 | <0.001 | HLA I T |
| RT | 91 | I91L | 18(18.4%) | 18(19.1%) | 0.8631 | 0.8797 | HLA I T |
| RT | 109 | P109S/Q | 60(60.2%) | 18(19.1%) | <0.001 | <0.001 | HLA I T |
| RT | 118 | T118N/A | 0 | 10 | <0.001 | <0.001 |  |
| RT | 121 | N121K/I/T | 3(3.1%) | 0 | 0.2466 | 0.2780 | HLA I T |
| RT | 122 | F122I | 68(68.4%) | 92(97.9%) | <0.001 | <0.001 | HLA I T |
| RT | 124 | Y124H | 0 | 18(19.1%) | <0.001 | <0.001 | HLA I T |
| RT | 126 | H126Y | 12(12.2%) | 19(20.2%) | 0.1259 | 0.1467 | HLA I T |
| RT | 128 | T128A | 18(18.4%) | 1(1.1%) | <0.001 | <0.001 |  |
| RT | 134 | D134E | 15(15.3%) | 14(14.9%) | 0.960 | 0.960 |  |
| RT | 135 | T135S | 29(29.6%) | 0 | <0.001 | <0.001 |  |
| RT | 139 | N139H | 1(1.0%) | 13(13.8%) | <0.001 | <0.001 | HLA I T |
| RT | 145 | L145M | 0 | 28(29.8%) | <0.001 | <0.001 | HLA I T |
| RT | 163 | I163V | 0 | 14(14.9%) | <0.001 | <0.001 | HLA I T |
| RT | 213 | S213T | 0 | 6(6.4%) | 0.0122 | 0.0158 | HLA I T |
| RT | 223 | S223A | 3(3.1%) | 55(58.5%) | <0.001 | <0.001 |  |
| RT | 224 | I224V | 4(4.1%) | 67(71.3%) | <0.001 | <0.001 |  |
| RT | 253 | V253R/I | 3(3.1%) | 20(21.3%) | <0.001 | <0.001 |  |
| RT | 263 | E263D | 0 | 36(38.3%) | <0.001 | <0.001 | HLA I T |
| RT | 267 | L267H/Q | 35(35.7%) | 90(95.7%) | <0.001 | <0.001 | HLA I T |
| RT | 269 | I269L | 37(37.8%) | 73(77.7%) | <0.001 | <0.001 | HLA I T |
| RT | 278 | V278I | 18(18.4%) | 62(66.0) | <0.001 | <0.001 | HLA I T |
| FDR:P-values were adjusted using the Benjamini-Hochberg method to control the false discovery rate (FDR) | | | | | | | |

**Table S5** Amino acid variations in “a” epitope (aa124–aa147)

|  | Genotype C | | | Genotype B/I | | |
| --- | --- | --- | --- | --- | --- | --- |
| Amino acid variations | HBeAg positive  （n=99） | HBeAg negative  （n=94) | *p* value | HBeAg positive  n=28 | HBeAg negative  n=68 | *p* value |
| C124S | 0 | 0 | - | 0 | 0 | - |
| T125M | 1 | 0 | - | 0 | 0 | - |
| I126T | 7 | 44 | <0.001 | 28 | 50 | 0.003 |
| I126S | 14 | 12 | 0.780 | 0 | 0 | - |
| G130N/R/E | 0 | 5 | 0.026 | 0 | 0 | - |
| T131I/N | 1 | 9 | - | 0 | 0 | - |
| M133T | 1 | 39 | <0.001 | 0 | 2 | - |
| F134K | 0 | 4 | 0.054 | 0 | 0 | - |
| G145R | 0 | 0 | - | 0 | 8 | 0.10 |

**Table S6** The patient numbers and mutation clone counts at drug-resistant mutation sites

| Position | Patients | Mutant clone/total clone | Clinic significance |
| --- | --- | --- | --- |
| rtA181T (SW172*) | No.19 | 6.3%(1/16) | LAM, LdT, ADF,TDF resistance |
| rtM204V (sI195M)  rtM204I (sW195S) | No.19 | 6.3%(1/16) | LAM, LdT resistance |
|  | No.11 | 7.1%(1/14) |  |
| rtN236T | No.18 | 78.3%(18/23) | ADV resistance |
| LAM, Lamivudine; LdT, Telbivudine; ADF, adefovir; ETV, Entecavir; TDF, Tenofovir. | | | |

**Table S7** Summary of deletion, insertion, and stop codon mutations in HBV quasispecies detected

| Patient ID (genotype) | positions in HBV genome | No. of mutant clones/total clones | Del/Ins aa patterns (aa positions of related region) |
| --- | --- | --- | --- |
| **HBeAg positive** | | | |
| P1(C) | nt2235A-T | 1/8 | Mutant LHBcAg at aa112 with stop codon |
|  | nt2397C-T | 1/8 | Mutant HBcAg at aa166 with stop codon |
|  | nt2421A-T | 1/8 | Mutant HBcAg at aa174 with stop codon |
|  | nt259G-A | 1/8 | Mutant LHBsAg at aa209 with stop codon |
| P2(C) | nt26-55 | 3/19 | Del LHBsAg at aa 132-141, Pol at aa 312-322 |
|  | nt11-55 | 15/19 | Del LHBsAg at aa 127- 141, Pol at aa 307-322 |
|  | nt2024 | 1/19 | Del frameshift PreC/C at aa 42with stop codon |
|  | nt1896G-A | 1/19 | PreC/C W28* |
|  | nt2455C-T | 6/19 | Mutant HBcAg at aa182 with stop codon |
| P7(C) | nt3066-3125 | 1/13 | Del LHBsAg aa73-93, Pol aa254-273 |
|  | nt47-101 | 1/13 | Del LHBsAg aa139-157, Pol aa319-337 and frameshift with stop codon |
|  | nt44-112 | 1/13 | Del LHBsAg aa138-160, Pol aa318-341 frameshift with stop codon |
|  | nt29-103 | 1/13 | Del LHBsAg aa133-157, Pol aa312-336 |
|  | nt1756-1744 | 4/13 | Del HBx aa128-134 |
|  | nt2528G-A | 1/13 | Mutant Pol at aa74 with stop codon |
|  | nt201 C-T | 1/13 | Mutant LHBsAg at aa190 with stop codon |
|  | nt1983C-T | 1/13 | Mutant HBcAg at aa 28 with stop codon |
| **HBeAg negative** | | | |
| P10(C) | nt1896G-A | 7/7 | PreC/C W28* |
|  | nt2345T-A | 1/7 | Mutant Pol at aa13 with stop codon |
|  | nt3207G-A | 7/7 | Mutation occurred in the start codon of Pres2 |
| P11(C) | nt29-55 | 11/13 | Del PreS1 aa133-141, del PreS2 aa14-22，del Pol aa312-320 |
|  | nt2982 C-T | 1/13 | Mutation occurred in Pol at aa226 with stop codon |
|  | nt3100-3108 | 1/13 | Del PreS1 aa84-86, Del Pol aa265-267 |
|  | nt1896G-A | 13/13 | PreC/C W28* |
|  | nt3206-nt3207 | 1/13 | Mutation occurred in the start codon of Pres2 |
| P12(C) | nt3-51 | 1/21 | Del LHBsAg aa124-139, del preS2 aa6-21, del Pol aa305-320 |
|  | nt8-52 | 2/21 | Del LHBsAg aa125-140, del preS2 aa6-21, del Pol aa306-321 |
|  | nt2849-2866 | 3/21 | Del PreS1 at start codon, Del Pol at aa181-186 |
|  | nt1575C-T | 1/21 | Mutant Pol at aa648 with stop codon |
|  | nt2560A-T | 1/21 | Mutant Pol at aa85 with stop codon |
|  | nt3086C-T | 1/21 | Mutant LHBsAg at aa80 with stop codon |
|  | nt1896G-A | 21/21 | PreC/C W28* |
|  | nt1814-1816 | 1/21 | Del ATG HBcAg start codon |
| P13(C) | nt223A-T | 1/22 | Mutant Pol at aa 378 with stop codon |
|  | nt361T-A | 1/22 | Mutant LHBsAg with at aa 243 with stop codon |
|  | nt1896G-A | 3/22 | PreC/C W28* |
| P14(C) | nt29-52 | 4/20 | Del LHBsAg aa133-140,Pre S2 aa14-21, Pol aa313-321 |
|  | nt2851-3060 | 2/20 | Del PreS1 aa2-72, Pol aa183-253 |
|  | nt2891-2956 | 2/20 | Del Pres1 aa15-36, Pol aa195-216 |
|  | nt366G-A | 1/20 | Mutant Pol at aa425 with stop codon |
|  | nt695C-T | 1/20 | Mutant LHBsAg at aa355 with stop codon |
|  | nt2464G-A | 1/20 | Mutant Pol at aa52 with stop codon |
|  | nt3205A-G | 4/20 | Mutant PreS2 at start codon |
| P15(C) | nt2849-2866 | 5/13 | Del LHBsAg start codon, Del Pol aa181-186 |
|  | nt1896G-A | 7/13 | PreC/C W28* |
|  | nt1023 C | 3/13 | Del and frameshift Pol at aa654 with stop codon |
| P16(C) | nt1631-1642 | 12/12 | Inserted into the HBxAg region with ‘CAGGAACCTGCC’ bases at 1631-1642 |
|  | nt1071T-A | 1/12 | Mutation occurred in Pol at aa660 with stop codon |
|  | nt1510C-T | 1/12 | Mutation occurred in Pol at aa807 with stop codon |
|  | nt1896G-A | 12/12 | PreC/C W28* |
|  | nt3207G-A | 1/12 | Mutation occurred in the start codon of Pres2 |
| P17(B) | nt3100-3108 | 13/17 | Del PreS1 aa 84-86, Pol aa265-268 |
|  | nt3208C-T | 3/17 | LHBsAg Q121* |
|  | ntG1896A | 17/17 | PreC/C W28* |
|  | nt2444C-T | 12/17 | Mutant HBcAg aa182 with stop codon |
|  | nt349T-A | 1/17 | Mutant LHBsAg aa239 with stop codon |
| P18(B) | nt41-43 | 1/23 | LHBsAg R137* |
|  | nt608C-A | 1/23 | Mutant Pol aa325 with stop codon |
|  | nt1896G-A | 23/23 | PreC/C W28* |
